# Comparative Metabolomics to Unravel the Biochemical Mechanism Associated with Rancidity in Pearl Millet (*Pennisetum glaucum* L.)

**DOI:** 10.3390/ijms252111583

**Published:** 2024-10-29

**Authors:** Kalenahalli Yogendra, Hemalatha Sanivarapu, Tejaswi Avuthu, Shashi Kumar Gupta, Priyanka Durgalla, Roopa Banerjee, Anitha Raman, Wricha Tyagi

**Affiliations:** International Crops Research Institute for the Semi-Arid Tropics, Hyderabad 502324, India; yogendra.kalenahalli@icrisat.org (K.Y.); hemalatha.icrisat@gmail.com (H.S.); tejusmedicine.96@gmail.com (T.A.); shashikumar.gupta@icrisat.org (S.K.G.); priyanka.durgalla@icrisat.org (P.D.); roopa.banerjee@icrisat.org (R.B.); anitha.raman@icrisat.org (A.R.)

**Keywords:** biochemical resistance, climate resilient, fatty acids, flavonoids, metabolomics, rancidity

## Abstract

Despite being a highly nutritious and resilient cereal, pearl millet is not popular among consumers and food industries due to the short shelf-life of flour attributed to rapid rancidity development. The biochemical mechanism underlying rancidity, a complex and quantitative trait, needs to be better understood. The present study aims to elucidate the differential accumulation of metabolites in pearl millet that impact the rancidity process. Metabolite profiling was conducted on ten pearl millet genotypes with varying levels of rancidity—comprising high, low, and medium rancid genotypes—utilizing liquid chromatography and high-resolution mass spectrometry (LC-HRMS) at different accelerated ageing conditions. Through non-targeted metabolomic analysis, crucial metabolites associated with rancidity were identified across various biochemical pathways, including fatty acids, glycerophospholipids, sphingolipids, glycerol lipids, flavonoids, alkaloids, and terpenoids. Notably, metabolites such as fatty aldehydes, fatty alcohols, fatty esters, fatty acyls, fatty esters, and fatty amides were significantly elevated in high rancid genotypes, indicating their involvement in the rancidity process. These fatty acids-related metabolites further break down into saturated and unsaturated fatty acids. Four key fatty acids—stearic, palmitic, linoleic and linolenic acid—were quantified in the ten pearl millet genotypes, confirming their role in rancidity development. This investigation promises novel insights into utilizing metabolomics to understand the biochemical processes and facilitate precision breeding for developing low-rancidity pearl millet lines.

## 1. Introduction

Globally, moderate food insecurity has resulted in hunger, as recent Food and Agriculture Organization data highlight, indicating a rise in undernourished individuals to 811 million by 2020 [1]. Micronutrient deficiencies, notably iron (Fe) and zinc (Zn) pose significant health risks, particularly among underprivileged and resource-poor people in semi-arid tropical regions worldwide. Pearl millet (*Pennisetum glaucum* L.), a nutritious staple cereal, boasts a rich profile of carbohydrates, proteins, fats, polyphenols, dietary fibres, vitamins, and essential micronutrients like Fe and Zn [2,3]. Although the flour derived from pearl millet offers nutritional benefits, it encounters challenges due to undesirable off-flavours. A mousy, acidic odour forms shortly after grinding, leading to rancidity, which lowers its acceptance in both the food industry and among consumers [4,5]. This adds to consumers’ drudgery as pearl millet has to be ground fresh before use. To combat rancidity, efforts have focused on developing post-harvest processing and pre-milling techniques to deactivate the biological components that accelerate rancidity. However, these mechanical and physicochemical methods might adversely affect the nutritional quality [4,5]. Therefore, tackling this issue remains challenging, but genetic enhancement can extend flour’s shelf life by addressing rancidity.

Rancidity in pearl millet has been associated with several contributing factors. These include the presence of volatile compounds [6] and changes in lipid composition due to hydrolytic breakdown [7]. Additionally, oxidative alterations in unsaturated fatty acids [8], increased peroxidase activity [9], and enzymatic modifications in C-glycosyl flavones [10,11] also play a role. A significant positive correlation exists between phenols, C-glycosyl flavones, peroxidase activity, and rancidity in pearl millet [12]. The enzyme lipase facilitates the breakdown of triacylglycerols into di- and monoglycerides, glycerol, and free fatty acids. Enzyme activity and yeast complementation assays for triacylglycerol-lipase (TAG lipase) were previously conducted in our lab using the selected pearl millet genotypes, providing evidence of the role of TAG lipase in the rancidity process [5]. Additionally, lipoxygenase acts on polyunsaturated fatty acids like linoleic, linolenic, and oleic acids. Primary oxidation results in the formation of hydroperoxides, which subsequently lead to secondary oxidation products, such as aldehydes and ketones, that contribute to off-flavours and odours [13]. Considering this, a comprehensive understanding of the chemical and biochemical factors involved in developing rancidity still needs to be improved. Once understood, the genetic mechanism underlying this variability in grains, if available, will help develop hybrids and varieties with reduced rancidity, enhancing the efficient utilization of nutrient-rich pearl millet in the food industry.

Metabolomics is crucial in deciphering the biochemical processes that determine grain quality. This approach explores plant metabolic networks and related gene functions by offering insights into metabolic pathways and identifying biomarkers. It involves the comprehensive qualitative and quantitative analyses of small molecule compounds within organisms. This helps reveal the complex processes that influence grain quality [14]. In pearl millet, solid-phase microextraction gas chromatography-mass spectrometry (SPME-GC-MS) was used for the identification of volatile organic compounds belonging to acid, alcohol, aldehyde, alkane, and ester groups across varying storage periods and temperatures to understand the rancidity process [6]. In addition, aldehyde estimation and levels of fatty acids, as well as primary and secondary oxidation (1-octen-3-ol and 2-pentyl furan), have been used to discriminate high, low, and medium rancid genotypes [5]. Notably, the data from these studies exclusively focused on volatile organic compounds through GC-MS, which poses certain limitations in detecting the broad spectrum of metabolites. Non-targeted metabolomics distinguishes itself through its impartial, systematic, and accurate identification of various metabolites in pearl millet, accurately reflecting the grain’s metabolic profile. This suggests a need to explore the application of non-targeted metabolomics using liquid chromatography high-resolution mass spectrometry (LC-HRMS) in the context of pearl millet.

The current study was designed to explore the biochemical mechanisms underlying pearl millet’s susceptibility to rancidity. Previous research on 56 diverse pearl millet genotypes suggests that the acid value (AV) and peroxide value (PV) serve as biochemical indicators for categorizing pearl millet genotypes into low, medium, and high rancidity categories [15]. Following the same methodology, we classified a subset of ten pearl millet hybrid parental lines into low, medium, and high rancidity groups to identify metabolites associated with the development of the rancidity process. To do this, we investigated variations in metabolite type and concentrations through liquid chromatography high-resolution mass spectrometry (LC-HRMS) under different storage conditions during accelerated ageing. Subsequently, the candidate metabolites were mapped onto metabolic pathways to understand their role in the rancidity process, and four such candidates were validated using targeted metabolite analysis. This metabolomic approach identifies candidate metabolites across contrasting pearl millet genotypes with specific rancidity patterns in metabolic pathways.

## 2. Results

### 2.1. Differential Accumulation of Metabolites in Flours of Pear Millet Genotypes

Metabolite profiles were examined in the flours of pearl millet genotypes with varying degrees of rancidity (low, medium, and high) at 0, 2, 5, 10, and 24 days following accelerated ageing, employing LC-HRMS analysis. Across all replicates, 10,007, 4183, 2644, 2005, and 4311 consistent peaks of monoisotopic masses were identified at the respective time points. Subsequently, a comparison of metabolites among the different rancidity levels was conducted, serving as the basis for further analysis (Tables S1–S5).

**Figure 1:**
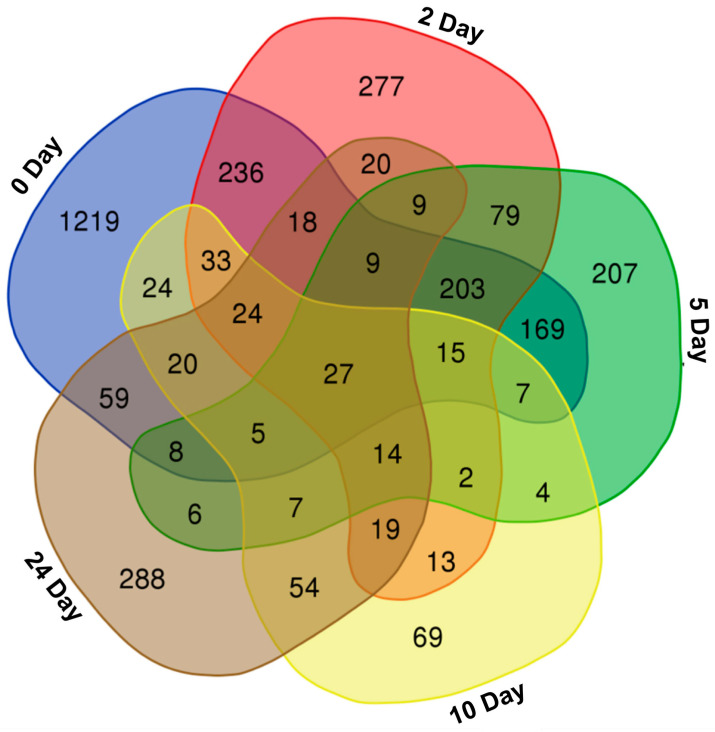
Venn diagram showing the differentially accumulated metabolites in ten pearl millet genotypes at 0, 2, 5, 10, and 24 days of accelerated ageing. The differentially accumulated metabolites were filtered using the following criteria: *p* value < 0.05 and log_2_ fold change > 1. Venn diagram was generated using the Draw Venn Diagram (https://bioinformatics.psb.ugent.be/webtools/Venn/, accessed on 15 January 2024) online tool.

**Figure 2:**
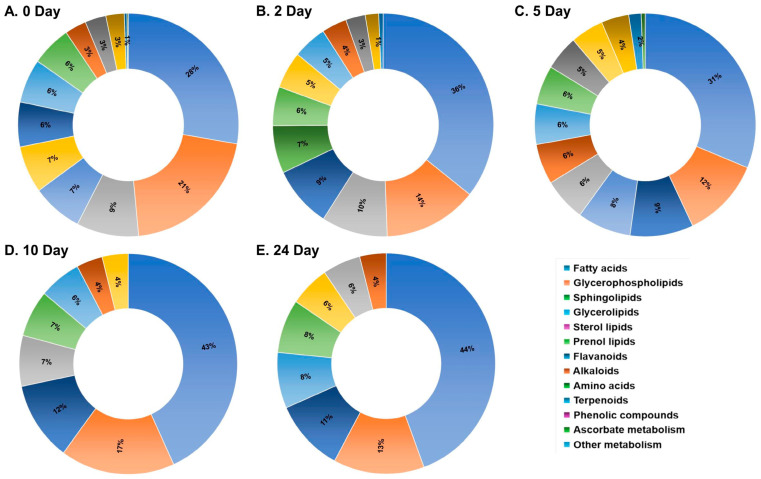
The percent distribution of the differentially accumulated metabolites observed in ten pearl millet genotypes at 0, 2, 5, 10, and 24 days of accelerated aging. The differentially accumulated metabolites were filtered using the following criteria: *p* value < 0.05 and log_2_ fold change > 1, and pathways were identified using the KEGG mapper. The percentages are calculated based on the number of significant metabolites in each category among the total number of significant metabolites obtained at that time point.

Differentially accumulated metabolites across ten pearl millet genotypes at the time points 0, 2, 5, 10, and 24 days were analyzed. Venn diagrams were created to compare the metabolites identified in the low, medium, and high rancidity genotypes (Figure 1). The largest number of metabolites showing differential accumulation was observed at day 0 (2076), followed by day 2 (998), day 5 (771), day 24 (587), and day 10 (337). A greater number of unique metabolites were detected during the early stages of rancidity (1219 at day 0), with a decline as the storage period increased (Tables S6–S10). Additionally, all differentially accumulated metabolites were classified into twelve distinct categories, and their percentage distribution was calculated (Figure 2).

### 2.2. Classification of Pearl Millet Genotypes Based on Principal Component Analysis (PCA) and Partial Least Squares-Discriminant Analysis (PLS-DA)

**Figure 3:**
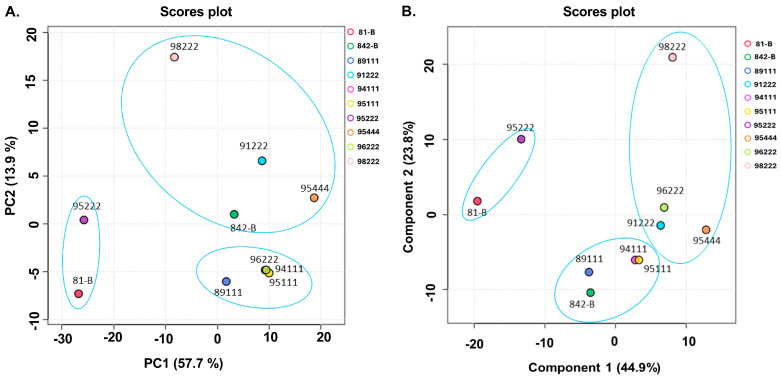
Overview of the multivariant analysis in pearl millet genotypes: (**A**) principal component analysis (PCA) and (**B**) partial least square-discriminate analysis (PLS-DA) of significant metabolites (*p* < 0.05) from low (ICMB89111, ICMB94111, ICMB95111, and ICMB95222), medium (81B and ICMB96222), and high rancid (842B, ICMB98222, ICMB95444, and ICMB91222) pearl millet genotypes at 0 days of accelerated ageing. The circle clearly shows the three distinct categories of low, medium, and high rancid pearl millet genotypes.

Principal component analysis (PCA) plots were generated to evaluate the similarities and differences among pearl millet genotypes with low, medium, and high rancidity at each time point (Figure 3). The results showed that the genotypes tended to cluster according to their rancidity categories, remaining distinct from one another, except for ICMB95222 and ICMBP96222 on day 0 (Figure 3A). The first two principal components (PC1 and PC2) together explained over 71% of the total variance in the dataset, with PC1 accounting for 57.7% and PC2 for 13.9%. In contrast, PC3 and higher components explained only a small fraction of the variance. However, this trend was not always consistent across other storage durations, even though the genotypes still formed three distinct clusters (Figures S1–S4). To further highlight differences between the low, medium, and high rancidity genotypes, Partial Least Squares-Discriminant Analysis (PLS-DA) was performed (Figure 3B and Figures S1–S4). The PLS-DA results reinforced the PCA findings, showing a clear separation between the genotypes at all time points and indicating differences in metabolite accumulation levels.

### 2.3. Pathway Enrichment Analysis

**Figure 4:**
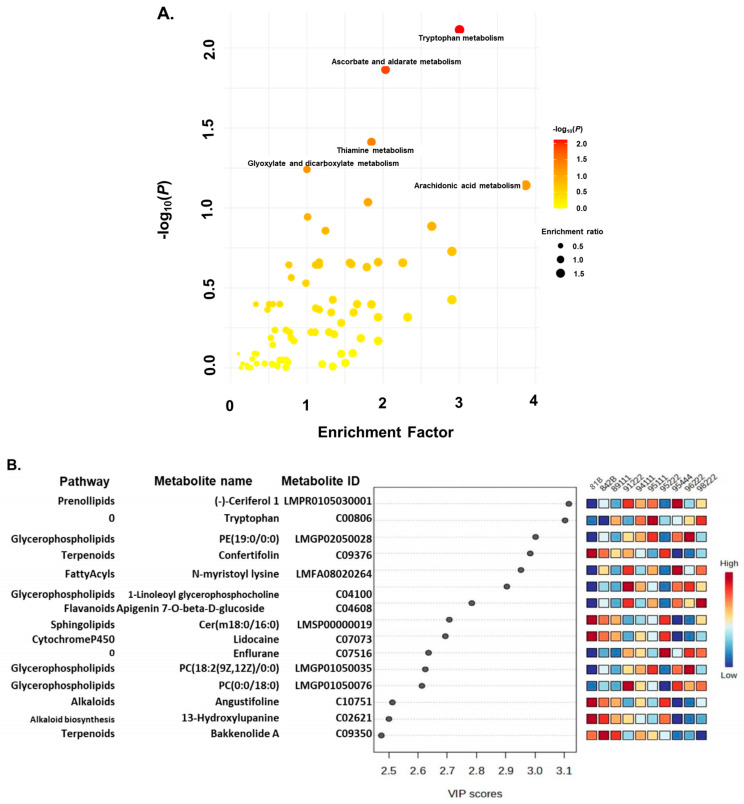
Pathway enrichment analysis (**A**) and variable importance in projection (VIP) scores (**B**) of metabolomics data from pearl millet genotypes at 0 days of accelerated ageing: (**A**) Dot plot of the enrichment analysis shows the significant pathways in red colour. The size of the circles per metabolite set represents the enrichment ratio (black dots) and the colour represents the *p* value. (**B**) VIP score indicates the top important metabolites (name and ID along with pathway indicated on the left) contributing to the separation of metabolic profiles in low (ICMB89111, ICMB94111, ICMB95111, and ICMB95222), medium (81 B and ICMB96222), and high rancid (842 B, ICMB98222, ICMB95444, and ICMB91222) pearl millet genotypes (top right). The relative abundance of metabolites is indicated by a coloured scale from blue to red, representing the low and high, respectively.

Statistically significant pathways (*p* < 0.05) were identified from low, medium, and high rancid genotypes at intervals of 0, 2, 5, 10, and 24 days post-accelerated ageing (Figure 4A and Figures S5–S8). The analysis revealed that specific pathways such as tryptophan, ascorbate and aldarate metabolism, thiamine metabolism, glyoxylate, dicarboxylate metabolism, and arachidonic acid metabolism were notably prevalent in the 0-day samples. However, as rancidity progressed during storage, there was a shift toward the biosynthesis of unsaturated fatty acids, α-linolenic acid metabolism, linoleic acid metabolism, arachidonic acid metabolism, flavone and flavanol biosynthesis, and sphingolipid metabolism across storage conditions.

Additionally, we computed the VIP values from low, medium, and high rancid genotypes at intervals of 0, 2, 5, 10, and 24 days following accelerated ageing. This analysis revealed that metabolites such as tryptophan, PC(19:0/0-0), 1-linoleoyl glycerophosphocholine, apigenin 7-O-beta-D-glucoside, cer(m18:0/16:0), PC(18:2(9Z,12Z)/0:0), and PC(0:0/18:0) exhibited differential accumulation specifically in the 0-day samples (Figure 4B). Moreover, we identified metabolites with differential accumulation across other time points based on their VIP values (Figures S5–S8).

### 2.4. Identification of Metabolites Associated with Rancidity

A total of 2076, 998, 771, 337, and 587 metabolites displaying differential accumulation were identified in pearl millet genotypes after 0, 2, 5, 10, and 24 days of accelerated ageing, respectively. Within these metabolites, 41, 214, 36, 35, and 40 were significantly accumulated in high, low, and medium rancid genotypes, respectively (Tables S1–S5). These metabolites span various chemical groups, including fatty acids, flavonoids, alkaloids, terpenoids, and phenolic compounds. Notably, fatty acid categories such as glycerophospholipids, sphingolipids, glycerol lipids, sterol lipids, and prenol lipids, collectively, encompassed over 50% of the identified metabolites.

**Figure 5:**
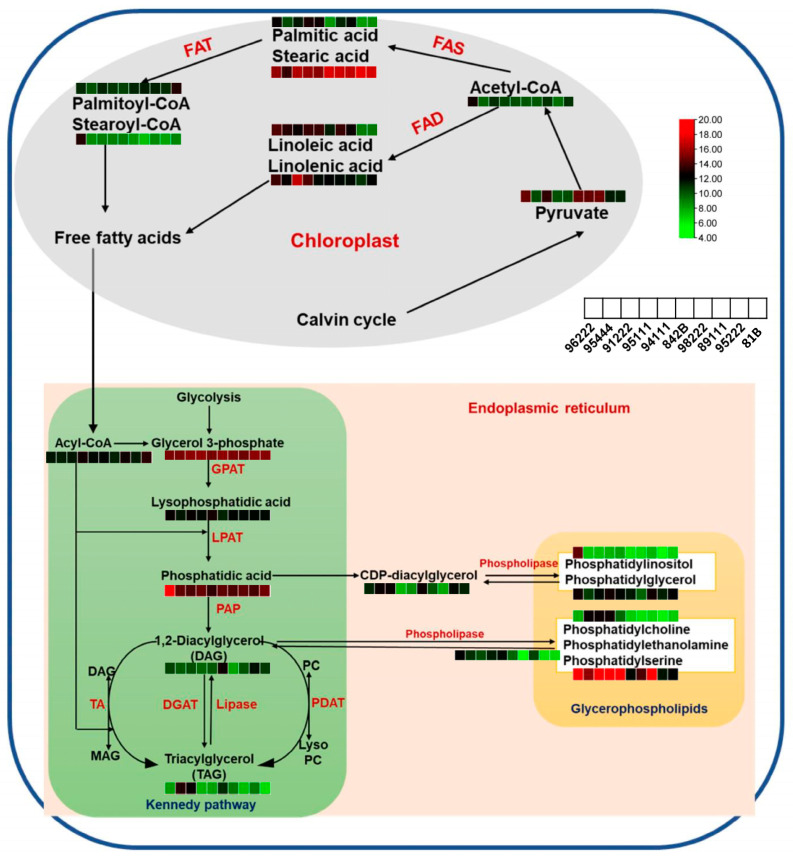
Overview of the triacylglycerides metabolic pathway showing the differentially accumulated metabolites in low (ICMB89111, ICMB94111, ICMB95111, and ICMB95222), medium (81 B and ICMB96222), and high rancid (842 B, ICMB98222, ICMB95444, and ICMB91222) pearl millet genotypes. The enzymes are FAS, fatty acid synthase; FAT, fatty acid thioesterase; FAD, fatty acid desaturase; ACS, acyl-CoA synthetase; GPAT, glycerol 3-phosphate acyltransferase; LPAT, lysophosphatidic acid acyltransferase; PAP, phosphatidic acid phosphatase; DGAT, diacylglycerol acyltransferase; TA, reversible transacylase and PDAT, phosphatidylcholine: diacylglycerol acyltransferase and two organelles are mentioned in red color fonts. The log_2_ fold change abundance values of the intermediate metabolites involved in various steps of the pathway obtained across the 10 pearl millet lines (mid-right) are represented as heatmaps (red and green indicating high and low abundance values, respectively).

Some of the important metabolites involved in rancidity (Figure 5) were as follows: 1. Fatty acid and conjugates-(2E)-tetracos-2-enoic acid, 13,16-docosadienoic acid, 3,4-dihydroxy-4-methylhexadecanoic acid, 15-hydroxy-pentacosanoic acid, 18-fluoro-oleic acid, 18-fluoro-9E-octadecenoic acid, and 2,14-dimethyl-octadecanoic acid; 2. Fatty acyls-8,11-nonadecadiene, 2-methyl-(Z)-7-octadecene, and 3Z,6Z,9Z-Nonadecatriene; 3. Fatty alcohols-iso-1,2-heptadecanediol and 1,2-tricosanediol; 4. Fatty esters-undecanoylcholine, arachidyl stearate, 4-methyl-3-heptyl palmitate, erectusfuranone B and (9Z,12Z)-3-hydroxyhexadecadienoyl-carnitine, 5. Fatty amides-Undeca-2E,4E-dien-8,10-diynoic acid isopentylamide, Linoleyl hydroxamic acid, N-(3-carboxypropanoyl)-N-hydroxyputrescine, and isobungeanool; 6. Glycerophospholipids: PG (14:1(9Z)/14:1(9Z)), PC(20:5(5Z,8Z,11Z,14Z,17Z)/18:3 (6Z,9Z,12Z)), PC (15:0/22:1(11Z)) and PIM1(19:2(9Z,12Z)/18:1(9Z)); 7. Sphingolipids: SM(d17:1/24:1), Cer (d14:2(4E,6E)/18:0(2OH)), and dehydrophytosphingosine; 8. Glycerolipids: DG (12:0/17:2(9Z,12Z)/0:0)[iso2], DG (12:0/17:1(9Z)/0:0)[iso2], TG (18:2(9Z,12Z)/22:1(13Z)/22:3(10Z,13Z,16Z))[iso6], and TG (15:1(9Z)/15:1(9Z)/16:0)[iso3]; 9. Prenol lipids: fucoxanthinol 3-linoleate 3′-gondoate, and all-trans-retinyl linolate; 10. Flavanoids: isorhamnetin 3-rhamnosyl-(1->6)-[rhamnosyl-(1->2)-(3‴-(E)-p-coumaroylgalactoside]-7-rhamnoside, isoliquiritigenin 2′-glucosyl-(1->4)-rhamnoside, kaempferol 3″2″-p-coumaryl-alpha-L-arabinopyranoside), and isorhamnetin 3″6″-malonylglucoside).

### 2.5. Validation of Key Metabolites Implicated in the Process of Rancidity

**Figure 6:**
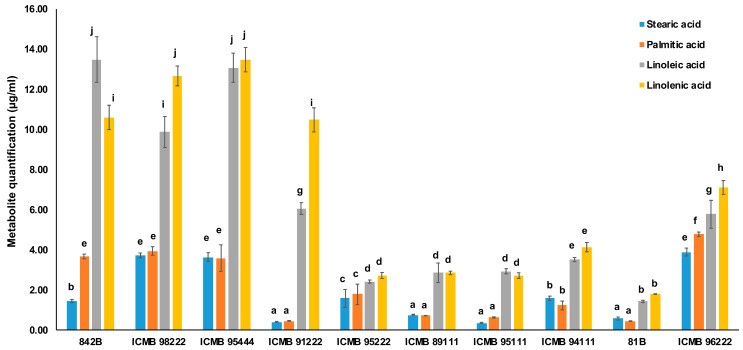
Quantifying four key fatty acids (stearic, palmitic, linoleic acid and linolenic acid) in ten pearl millet genotypes using the multiple reaction monitoring (MRM) technique in liquid chromatography–mass spectrometry (LC-MS) at day 0 of accelerated ageing. The Y-axis represents the mean values of the metabolite quantification (µg/mL), while the X-axis indicates genotypes. The results are representative of six independent samples, and values indicate mean ± standard error of replicates. The mean values followed by the same alphabet on top indicate no difference (Tukey’s Test, *p* < 0.05). All the mean values are statistically significant as determined using one-way ANOVA (*p* < 0.05).

The multiple reaction monitoring (MRM) technique in LC-MS was utilized for the quantification of four free fatty acids: palmitic acid (PA), stearic acid (SA), linoleic acid (LIA), and linolenic acid (LNA) from high, low, and medium rancid pearl millet genotypes in samples collected at 0 days (Figure 6). Among these, two saturated fatty acids, stearic acid, and palmitic acid, exhibited higher concentrations in high rancid genotypes (SA: 0.44–3.71 µg/mL and PA: 0.45–3.94 µg/mL), followed by medium (SA: 0.57–3.88 µg/mL and PA: 0.44–4.77 µg/mL) and low rancid (SA: 0.36–1.58 µg/mL and PA: 0.62–1.77 µg/mL) genotypes. Additionally, two unsaturated fatty acids showed elevated levels in high rancid genotypes (LIA: 6.05–13.48 µg/mL and LNA: 10.47–13.48 µg/mL), followed by medium (LIA: 1.42–5.77 µg/mL and LNA: 1.79–7.10 µg/mL) and low rancid (LIA: 2.41–3.51 µg/mL and LNA: 2.71–4.13 µg/mL) genotypes. 

The correlation analysis between acid value and the composition of saturated and unsaturated fatty acids in low rancid genotypes indicates a positive correlation with saturated fatty acids (Figure S9A). Conversely only linolenic acid demonstrates a negative correlation in medium rancid genotypes. However, in high rancid genotypes, both palmitic and linoleic acid exhibit negative correlations.

Furthermore, the correlation between peroxide value and the composition of saturated and unsaturated fatty acids reveals that stearic acid, linoleic acid, and linolenic acid are negatively correlated in both low and high rancid genotypes (Figure S9B). Similarly, linolenic acid displays a negative correlation in medium rancid genotypes, like its correlation with acid value. Notably, the extent of the negative correlation of linolenic acid with PV amongst the rancidity groups is low > medium > high, with high rancid lines showing maximum negative correlation.

## 3. Discussion

Although a nutritious grain, pearl millet flour is susceptible to rancidity, resulting in unpleasant odours and flavours that diminish its quality [4,5]. The emergence of off-flavours in pearl millet flour during storage is a significant barrier to consumer acceptance. This long-standing issue has hindered the utilization of pearl millet in the food industry and its adoption by urban populations. While various methods, such as hydrothermal treatment and combinations of techniques, have shown some effectiveness in reducing rancidity, their economic viability on a large scale may be impractical for rural agricultural communities [16]. Enhanced shelf life through rigorous treatments implies that enzymatic processes likely generate off-flavour compounds in pearl millet flour [17]. Therefore, understanding the complex biochemical mechanisms behind rancidity is essential for developing practical solutions to this challenge.

Metabolomics, as a systems biology approach, is crucial in unravelling the biochemical mechanisms underlying grain quality. It achieves this by offering insights into metabolic pathways, pinpointing biomarkers, and conducting thorough qualitative and quantitative analyses of all inherent small molecule compounds within organisms. This approach delves into plant metabolic networks and associated gene functions, shedding light on the complex processes involved in grain quality [14]. Metabolomics has found widespread application in examining crop nutrients and assessing quality across major crops, such as rice and wheat [18,19,20]. Previous studies involving pearl millet genotypes using GC-MS analysis, coupled with multivariate data analysis methods like PCA and PLS-DA, have been utilized for categorizing volatile organic compounds across varying storage periods and temperatures [6]. While several metabolomics techniques have been documented, non-targeted metabolomics analysis stands out for its unbiased, systematic, and precise identification of diverse metabolites, mirroring the grain’s metabolic profile. However, applying non-targeted metabolomics employing LC-HRMS still needs to be explored in the context of pearl millet.

### 3.1. Rancidity in Pearl Millet Is Associated with the Presence of Metabolites Derived from Fatty Acids

We observed differential fatty acid metabolites across pearl millet genotypes with differing degrees of rancidity: low, medium, and high. Significant accumulations of various fatty acid compounds were noted in the high rancidity genotypes compared to those with low and medium rancidity. Specifically, we detected increased levels of fatty acid conjugates such as 5-acetamidovalerate, (2E)-tetracos-2-enoic acid, 13,16-docosadienoic acid, 3,4-dihydroxy-4-methylhexadecanoic acid, 18-fluoro-oleic acid, 18-fluoro-9E-octadecenoic acid, and 2,14-dimethyl-octadecanoic acid. Additionally, fatty acyls, including 2-methyl-7R,8S-epoxy-octadecane, 8,11-nonadecadiene, and 2-methyl-(Z)-7-octadecene were elevated. Furthermore, fatty alcohols such as iso-1,2-heptadecanediol, 1,2-tricosanediol, and behenyl alcohol, along with fatty esters like undecanoylcholine, Linolenyl linolenate, (9Z,12Z)-3-hydroxyhexadecadienoylcarnitine, and FAHFA 18:1/32O(FA 32:1) (23Z-32-(9Z-octadecenoyloxy)dotriacontenoic acid) showed significant increases. Moreover, fatty amides such as undeca-2E,4E-dien-8,10-diynoic acid isopentylamide, anandamide (20:1, n-9), and N-hydroxy arachidonoyl amine were detected in higher concentrations. Additionally, fatty aldehydes such as 2-decene-4,6,8-triyn-1-al, heptadecanal, and 14S-Methyl-8E-hexadecenal were elevated in high rancidity genotypes compared to their counterparts. Previous research has established that during the rancidity process, long-chain fatty acids undergo breakdown into short-chain compounds [21]. Volatile organic compounds, including alcohols, hydrocarbons, aldehydes, ketones, esters, acids, benzene derivatives, heterocycles, and sulfur compounds, have been identified as biomarkers of rancidity in pearl millet during hydrolytic and oxidative rancidity [4,5,6,11]. These volatile chemicals serve not only as key markers of oil oxidation but also play a role in the development of rancidity in foods rich in polyunsaturated fats, such as seeds and flour [22,23].

Pearl millet grain, along with maize [24], stands out with its larger germ layer and elevated lipid content (5–7%), which is rich in unsaturated fatty acids [25]. When the whole grain is milled, the bran and germ layers break, activating endogenous lipases. These enzymes initiate the hydrolysis of stored lipids, leading to the release of free fatty acids (FFAs). Consequently, more significant metabolites from glycerolipids (TG(18:2(9Z,12Z)/22:1(13Z)/22:3(10Z,13Z,16Z))[iso6], TG(20:1(11Z)/22:3 (10Z,13Z,16Z)/22:3(10Z,13Z,16Z))[iso3], TG(15:1(9Z)/15:1(9Z)/16:0)[iso3], DG(12:0/17:1(9Z)/0:0)[iso2], DG(12:0/17:2(9Z,12Z)/0:0)[iso2], and 1-O-(2R-hydroxy-nonadecyl)-sn-glycerol), glycerophospholipids (PG(14:1(9Z)/14:1(9Z)), PC(20:5(5Z,8Z,11Z,14Z,17Z)/18:3(6Z,9Z,12Z)), PC(15:0/22:1(11Z)), PIM1(19:2(9Z,12Z)/18:1(9Z)), PS(22:2(13Z,16Z)/22:4(7Z,10Z,13Z,16Z)), PS(O-16:0/0:0), PE(P-16:0/0:0), PI(14:1(9Z)/0:0) and PG(P-16:0/0:0)) and sphingolipids (SM(d17:1/24:1), (4E,8E,d18:2) sphingosine, (4E,8E,10E-d18:3) sphingosine, Cer(d16:1/17:0), Cer(d16:1/20:0), Cer(d18:2(4E,8E)/14:0(2OH)), and Cer(t18:1(6OH)/28:0(2OH))) were observed in highly rancid genotypes. The primary reserves of lipids are triacylglycerols (TAGs), enclosed within oil bodies enveloped by phospholipid membranes. It is known that the aleuronic layer (made up of cells with phospholipids), with maturity and during storage, undergoes changes. Primarily, the phospholipids present in the membranes undergo decomposition into fatty acids, which is facilitated by phospholipid-degrading enzymes. These enzymes, such as phospholipases, acyl hydrolases, and lipid-oxidizing enzymes, significantly contribute to membrane degradation [26,27]. *Oryza officinalis* rice accessions with variations in *OsPLDα1* have lower transcript abundance and therefore are able to regulate the quality of rice bran oil [28]. Additionally, the natural variation observed in TAG lipase genes *PgTAGLip1* and *PgTAGLip2* contributes to reduced accumulation of free fatty acids in grains post-milling in pearl millet [5]. The recent report on the genome-wide identification of phospholipase genes in pearl millet suggests the potential role of at least five genes—*PgPLD-alpha1-1*, *PgPLD-alpha1-5*, *PgPLD-delta1-7a*, *PgPLA1-II-1a*, and *PgPLD-delta1-2a*—in addressing the issue of rancidity in pearl millet and improving grain quality trait [29]. Furthermore, the targeted estimation of unsaturated fatty acids (linoleic and linolenic acid) and saturated fatty acids (stearic acid and palmitic acid) across high, low, and medium rancid pearl millet genotypes highlights their involvement in the rancidity process. Similarly in oil palm fruit, free fatty acid levels significantly correlated with palmitic acid, stearic acid, myristic acid, and palmitoleic acid [30]. This suggests that producing free fatty acids as degraded products of triacylglycerides and phospholipids accelerates the rancidity process in pearl millet flour during storage. Fatty acid-related metabolites play a crucial role in mitigating rancidity in pearl millet flour, and their targeted manipulation in breeding programs provides a pathway to develop higher-quality varieties with more durable flour shelf-life. By concentrating on the relationship between fatty acid composition and stability at the grain level, breeders can cultivate pearl millet that satisfies consumer preferences.

### 3.2. Pearl Millet Rancidity Is Associated with the Presence of Flavonoid-Related Metabolites

Metabolites of the flavonoid pathway, present in diverse plant sources, are recognized for their antioxidative property. Nevertheless, their involvement in the rancidity process exhibits variability depending upon the particular flavonoid and the rancidity conditions. Flavonoids commonly engage in free radical scavenging and oxidative stress inhibition. Particularly in the early phases of rancidity, flavonoids can mitigate lipid oxidation initiation by neutralizing free radicals, thus postponing the onset of rancidity [31]. We observed a higher number of flavonoid-related metabolites such as 2-O,3-dimethylflaviolin, pelargonidin 3-rutinoside, pelargonidin 7-glucoside, dihydroformononetin 7-O-glucoside, isorhamnetin 3-(6″-malonylglucoside), 3,5,6,7,3′,4′,5′-heptamethoxyflavone, kaempferol 3-(2″-p-coumaryl-alpha-L-arabinopyranoside), and apigenin 7-O-beta-D-glucoside in low rancid genotypes compared to higher ones, suggesting a potential protective effect against rancidity. Nonetheless, under specific circumstances, flavonoids can exhibit pro-oxidant tendencies, particularly in the presence of transition metals or at elevated concentrations [32]. Furthermore, exposure to light and oxygen can trigger photochemical reactions in flavonoids, leading to the generation of reactive oxygen species that may initiate lipid oxidation and contribute to rancidity [33]. Consequently, our study observed an increase in the percentage of flavonoids under various accelerated ageing conditions during storage. Based on our results, we speculated that as our understanding of these compounds grows, we may be able to develop new strategies for using flavonoids to decrease the rate of rancidity process in pearl millet.

Further, incorporating metabolite profiling into pearl millet breeding programs offers a comprehensive strategy for improving grain quality in this climate resilience crop. By selectively targeting beneficial metabolites, breeders can lower flour rancidity while also addressing other key crop traits. A better understanding of pathway(s) and underlying gene(s) leading to the synthesis of these metabolites will result in a targeted molecular strategy for generating superior grain-quality pearl millet genotypes. This will lead to the development of more nutritious, market-ready varieties that cater to consumer preferences, and bolster farmers’ livelihoods.

## 4. Materials and Methods

### 4.1. Plant Genotypes and Storage Conditions

Ten hybrid parental lines developed at the International Crops Research Institute for the Semi-Arid Tropics (ICRISAT) in India underwent characterization for traits associated with rancidity, such as acid value and peroxide value [15]. These lines consist of low-rancidity ICMB89111, ICMB94111, ICMB95111, and ICMB95222, medium-rancidity 81 B and ICMB96222, and high-rancidity 842 B, ICMB98222, ICMB95444, and ICMB91222 lines. Originating from breeding efforts at ICRISAT from 1981 to 1998, these seed parental lines have served as progenitors for numerous popular pearl millet hybrids cultivated in India, showcasing considerable diversity in their pedigrees. This study leveraged the distinctive characteristics of these lines for further analysis.

The seeds were stored in vacuum-sealed pouches in a cold room prior to analysis. A 30 g portion from each sample was ground into a fine powder using a Cyclotec (Foss Analytical AB, Höganäs, Sweden) grinding mill. The resulting flours were evenly spread in lidless, food-grade trays (4 oz./118 mL) and subjected to accelerated storage conditions in incubator chambers set at 35 ± 0.1 °C and 75 ± 3% relative humidity. For metabolomics analysis, sub-sampling was carried out on the 0, 2, 5, 10, and 24 days of storage. The same time points were used for the estimation of AV and PV values with three biological replications, as mentioned previously [15].

### 4.2. Metabolite Extraction and LC-High-Resolution MS (LC-HRMS) Analysis

The experimental units consisted of flour samples of ten pearl millet genotypes (four each of low and high rancid and two medium rancid genotypes) with three replications, where the AV and PV were estimated as mentioned in Section 4.1. Metabolites were extracted from the flour samples using 80% aqueous methanol with 0.1% formic acid [34,35]. These were analyzed in positive ionization mode using the Waters Ultra Performance Liquid Chromatography system (UPLC-XEVO-G2XS-ESI-QTOF).

The LC-HRMS output files were converted to mzXML format. Data were processed using the interactive LC-MS data processing software MZmine-2.53 with the high-sensitivity peak detection algorithm XCMS centWave [36]. The observed masses and their relative abundance were imported; peaks that were inconsistent among replicates and those annotated as isotopes and adducts were excluded from further analyses.

Metabolites with *p* < 0.05 were considered differentially expressed. The corrected *p* value and log_2_ fold change were used as the threshold of significant differential expression. The significant metabolites were putatively identified in different databases: METLIN, plant metabolic network (PMN), LIPIDMAPS, and KEGG. Features detected from MS positive ionization modes were combined, and duplicate features were removed. Peak heights were normalized using the constant median method, and the normalized peak height data of unique features were exported, formatted, and uploaded to MetaboAnalyst 5.0, a robust tool for metabolomic data analysis [37]. It employed two key multivariate analysis techniques: principal component analysis (PCA) and partial least squares discriminant analysis (PLS-DA). Pathway enrichment analysis was conducted to reveal biological pathways significantly enriched with the differentially expressed metabolites, offering insights into underlying biological processes affected by experimental conditions. Variable importance in projection (VIP) scores from PLS-DA were utilized to assess the contribution of individual metabolites to group discrimination. In combination, these analyses provided a comprehensive framework for understanding the metabolomic changes associated with the experimental condition, pathway enrichment analysis, and variable importance in projection (VIP). Venn diagrams were generated using Draw Venn Diagram (https://bioinformatics.psb.ugent.be/webtools/Venn/, accessed on 15 January 2024).

### 4.3. Metabolomic Data Analysis

Given the ambiguity in categorizing ten pearl millet genotypes into high, low, and medium rancidity groups solely based on AV and PV values, a more insightful approach was adopted. Initially, metabolites obtained at various time points (0, 2, 5, 10, and 24 days) for all metabolites were classified into distinct chemical groups based on their pathways and biological classification. These groups encompassed fatty acids, glycerophospholipids, sphingolipids, glycerolipids, sterol lipids, prenol lipids, flavonoids, alkaloids, terpenoids, amino acids, phenolic compounds, and other metabolites.

Each metabolite class was then individually analyzed by taking the average of the three replications for each genotype and then computing the means for the high, low, and medium rancidity genotypes (four each of low and high rancidity and two medium rancidity genotypes as inferred by AV and PV values). Sample-based standard deviations were calculated for each genotype category to assess the dispersion of values from the mean. Standard errors of the estimated standard deviations provided insights into the precision of the sample mean. A constant parameter of 2.7, derived from a *t* value corresponding to a 98% confidence level and 10 degrees of freedom for a two-tailed test, was utilized to determine the range of critical values within each genotype category for all the metabolite classifications. All statistically significant metabolites across all classes were then filtered, ultimately pinpointing fatty acids and glycerophospholipids as the primary compounds significantly associated with rancidity in pearl millet.

These insights from metabolomics were then correlated with the genotypes’ AV and PV values to establish criteria for classifying genotypes into high, low, and medium rancidity groups. Fatty acid metabolites potentially responsible for rancidity in pearl millet were also identified. These identified fatty acid metabolites were further estimated using the multiple reaction monitoring (MRM) technique in LC-MS using a standard, as mentioned in the next section.

### 4.4. Estimation of Saturated and Unsaturated Fatty Acids Using LC-MS

The MRM technique in LC-MS was used to quantify four free fatty acids: palmitic acid (PA), stearic acid (SA), linoleic acid (LIA), and linolenic acid (LNA) (Table 1). Analytical-grade Sigma-Aldrich (St. Louis, MO, USA) reference standards were utilized. Initially, standard compounds were dissolved in methanol to prepare stock solutions with a concentration of 1000 mg/L, which were then stored at 4 °C. Subsequently, working standard solutions were prepared by diluting the stock solutions to a 500 ng/mL concentration. This procedure ensured the availability of fresh and precise standard solutions for each day of analysis.

#### 4.4.1. Extraction

Fatty acids were extracted from the flour samples at 0 days by shaking them with a 1:2 (*v*/*v*) mixture of chloroform and methanol (2 mL) at room temperature for 15 min. Phase separation was initiated by adding 0.5 mL of chloroform and 1.2 mL of water. The lower lipid layer was collected, and two additional extractions were performed with 1 mL of chloroform each time. After extraction, the lipid extracts were filtered and dried under vacuum conditions [5]. The resulting extract was dissolved and filtered through a 0.2 mm-pore PTFE membrane (PHENEX, Phenomenex, Torrance, CA, USA), and the resulting filtrate was utilized for LC-MS analysis.

#### 4.4.2. Quantification

The LC-MS analysis was performed using a Waters UPLC-XEVO-G2XS-ESI-QTOF system in the negative-ion mode. A lock spray interface ensures accurate mass measurement, with leucine-enkephalin as the lock-mass compound. Data acquisition was conducted using MassLynx v4.1 software, which Waters provides. To ensure a precise and reliable analysis of the samples under investigation [38], the Acquity UPLC BEH-C18 reversed-phase column (2.1 mm internal diameter and 100 mm length, with a particle size of 1.7 µm) was used for the analysis. The column temperature is maintained at 40 °C throughout the analysis to optimize the separation and retention of compounds in the sample during the chromatographic process. Buffer A—2 mM ammonium formate, buffer B—99% acetonitrile, and 1% 0.5 mM ammonium acetate were used with a flow rate of 0.3 mL/min, and the isocratic conditions were set to 60% buffer-B throughout the run. Samples were analyzed in MRM mode for the following fatty acids: LNA, LA, PA, and SA with the following parameters: capillary voltage—3 kV, Cone energy—15 eV, collision energy—25 eV, source temperature—120 °C and desolvation temperature—300 °C [39]. Calibration curves were generated using the peak area of m = z [M−H] ± 0.05 Da divided by the area of the internal standard. The mean values of the four metabolites were calculated from six independent samples, and standard errors were estimated. The significance of the metabolites was assessed using Tukey’s Test, with a *p* value threshold of <0.05. One-way ANOVA (*p* < 0.05) was used to identify the significant mean values across the genotypes.

## 5. Conclusions

In summary, this study integrates non-targeted and targeted metabolomics data to elucidate the biochemical processes involved in rancidity in pearl millet. The non-targeted metabolomics analysis highlighted the significant role of fatty acids and flavonoid-related compounds in the rancidity mechanism. After validation on a larger panel of genotypes, these free fatty acids linked to rancidity could be valuable biomarkers for routine testing of genotypes prone to rancidity, aiding pearl millet improvement programs. By incorporating metabolomics into pearl millet research, we can enhance our understanding and management of rancidity, ultimately improving this nutritious grain’s quality and shelf life. Additionally, the understanding of pathway(s) and underlying gene(s) leading to the synthesis of these metabolites will result in a targeted molecular strategy to generate superior grain-quality genotypes. Extending the shelf life of flour made from pearl millet offers significant opportunities for both primary and secondary processing industries, boosting market growth and increasing profits for small-scale farmers. The insights gained from this research will identify new targets for precision breeding in pearl millet to mitigate rancidity.

## Figures and Tables

**Table 1:** The saturated and unsaturated fatty acids, along with their molecular formulae, masses (Daltons (Da)), and retention time (minutes (min)), were used for the MRM technique in LC-MS.

Name	Molecular Formula	Exact Mass (Da)	Retention Time (min)	Observed Mass (Da)
Palmitic acid (PA, C16:0)	C_16_H_32_O_2_	256.24023	1.94	255.23 [M−H]
Stearic acid (SA, C18:0)	C_18_H_36_O_2_	284.27153	2.94	283.26 [M−H]
Linoleic acid (LIA, C18:2)	C_18_H_32_O_2_	280.24023	1.47	279.23 [M−H]
Linolenic acid (LNA, C18:3)	C_18_H_30_O_2_	278.22458	1.18	277.22 [M−H]

## Data Availability

The data supporting this study’s findings are available in Supplementary Figures S1–S9 and Supplementary Tables S1–S10 of this article.

## Supplementary Materials

The following supporting information can be downloaded at: https://www.mdpi.com/article/10.3390/ijms252111583/s1.

## References

[1] (2020). FAOSTAT. http://www.fao.org/faostat/en/#data.

[2] Mahendrakar M.D., Kumar S., Singh R.B., Rathore A., Potupureddi G., Kishor P.K., Gupta R., Srivastava R.K. (2019). Genetic variability, genotype× environment interaction and correlation analysis for grain iron and zinc contents in recombinant inbred line population of pearl millet [*Pennisetum glaucum* (L). R. Br.]. Indian J. Genet. Plant Breed..

[3] Srivastava R.K., Satyavathi C.T., Mahendrakar M.D., Singh R.B., Kumar S., Govindaraj M., Ghazi I.A. (2021). Addressing iron and zinc micronutrient malnutrition through nutrigenomics in pearl millet: Advances and prospects. Front. Genet..

[4] Goswami S., Asrani P., Ansheef Ali T., Kumar R.D., Vinutha T., Veda K., Kumari S., Sachdev A., Singh S.P., Satyavathi C.T. (2020). Rancidity matrix: Development of biochemical indicators for analysing the keeping quality of pearl millet flour. Food Anal. Methods.

[5] Aher R.R., Reddy P.S., Bhunia R.K., Flyckt K.S., Shankhapal A.R., Ojha R., Everard J.D., Wayne L.L., Ruddy B.M., Deonovic B. (2022). Loss-of-function of triacylglycerol lipases are associated with low flour rancidity in pearl millet [*Pennisetum glaucum* (L.) R. Br.]. Front. Plant Sci..

[6] Selvan S.S., Mohapatra D., Kate A., Kar A., Modhera B. (2023). Mapping and analysis of volatomes from pearl millet (*Pennisetum glaucum* L.) grains during different storage conditions with solid-phase microextraction–gas chromatography–mass spectrometry. Cereal Chem..

[7] Goswami S., Kumar R.R., Praveen S. (2019). Hydrolytic and oxidative decay of lipids: Biochemical markers for rancidity measurement in pearl millet flour. Omics Meet Plant Biochem. Appl. Nutr. Enhanc. One Health Perspect..

[8] Bhargavi H., Singh S.P., Goswami S., Yadav S., Aavula N., Shashikumara P., Singhal T., Sankar S.M., Danakumara T., Hemanth S. (2024). Deciphering the genetic variability for biochemical parameters influencing rancidity of pearl millet (*Pennisetum glaucum* LR Br.) Flour in a set of highly diverse lines and their categorization using rancidity matrix. J. Food Compos. Anal..

[9] Goyal P., Chugh L.K. (2014). Partial purification and characterization of peroxidase from pearl millet (*Pennisetum glaucum* [L.] R. Br.) grains. J. Food Biochem..

[10] Boncompagni E., Nielsen E., Sanogo M.D., Coghetto V., Temporiti M.E., Daglia M., Di Lorenzo A., Doria E. (2019). Anti-nutritional metabolites in six traditional African cereals. J. Food Nutr. Res..

[11] Ali A., Kumar R.R., Bansal N., Bollinedi H., Singh S.P., Satyavathi C.T., Praveen S., Goswami S. (2023). Characterization of biochemical indicators and metabolites linked with rancidity and browning of pearl millet flour during storage. J Plant Biochem Biotech..

[12] Deepak Y., Chhabra A., Laxman C., Behl R. (2010). Genotypic variability for rancidity and its association with morphological and biochemical traits in pearl millet [*Pennisetum Glaucum* (L.) R. Br.]. Ann. Biol..

[13] Grebenteuch S., Kanzler C., Klaußnitzer S., Kroh L.W., Rohn S. (2021). The formation of methyl ketones during lipid oxidation at elevated temperatures. Molecules..

[14] Hamany Djande C.Y., Pretorius C., Tugizimana F., Piater L.A., Dubery I.A. (2020). Metabolomics: A tool for cultivar phenotyping and investigation of grain crops. Agronomy.

[15] Mazumdar S., Gupta S., Banerjee R., Gite S., Durgalla P., Bagade P. Determination of variability in rancidity profile of select commercial pearl millet varieties/hybrids. Proceedings of the CGIAR Research Program on Dryland Cereals Review Meeting.

[16] De Vos R.C., Moco S., Lommen A., Keurentjes J.J., Bino R.J., Hall R.D. (2007). Untargeted large-scale plant metabolomics using liquid chromatography coupled to mass spectrometry. Nat. Protoc..

[17] Avuthu T., Sanivarapu H., Prasad K., Sharma N., Sudini H.K., Yogendra K. (2024). Comparative metabolomics analysis reveals secondary cell wall thickening as a barrier to resist *Aspergillus flavus* infection in groundnut. Physiol. Plant.

[18] Pluskal T., Castillo S., Villar-Briones A., Orešič M. (2010). MZmine 2: Modular framework for processing, visualizing, and analyzing mass spectrometry-based molecular profile data. BMC Bioinform..

[19] Pang Z., Chong J., Zhou G., de Lima Morais D.A., Chang L., Barrette M., Gauthier C., Jacques P.-É., Li S., Xia J. (2021). MetaboAnalyst 5.0: Narrowing the gap between raw spectra and functional insights. Nucleic Acids Res..

[20] Takahashi H., Suzuki H., Suda K., Yamazaki Y., Takino A., Kim Y.-I., Goto T., Iijima Y., Aoki K., Shibata D. (2013). Long-chain free fatty acid profiling analysis by liquid chromatography–mass spectrometry in mouse treated with peroxisome proliferator-activated receptor α agonist. Biosci. Biotechnol. Biochem..

[21] Hummel J., Segu S., Li Y., Irgang S., Jueppner J., Giavalisco P. (2011). Ultra performance liquid chromatography and high resolution mass spectrometry for the analysis of plant lipids. Front. Plant Sci..

[22] Padmaja P., Kalaisekar A., Venkateswarlu R., Shwetha S., Rao B.D., Tonapi V. (2023). Thermal treatment in combination with laminated packaging under modified atmosphere enhances the shelf life of pearl millet flour. Food Chem. Adv..

[23] Satyavathi C.T., Praveen S., Mazumdar S., Chugh L., Kawatra A. (2017). Enhancing Demand of Pearl Millet as Super Grain. http://www.aicpmip.res.in/Enhancing_Demand_of_Pearlmillet_as_Super_Grain.pdf.

[24] Chen Z., Chen H., Jiang Y., Wang J., Khan A., Li P., Cao C. (2020). Metabolomic analysis reveals metabolites and pathways involved in grain quality traits of high-quality rice cultivars under a dry cultivation system. Food Chem..

[25] Shi Q., Wang R., Lu W., Zhu J., Zhang H., Xiong Q., Zhou N. (2024). Metabolomics analysis of variation in grain quality of high-quality japonica rice. Agronomy.

[26] Shi T., Zhu A., Jia J., Hu X., Chen J., Liu W., Ren X., Sun D., Fernie A.R., Cui F. (2020). Metabolomics analysis and metabolite-agronomic trait associations using kernels of wheat (*Triticum aestivum*) recombinant inbred lines. Plant J..

[27] Heiniö R., Lehtinen P., Oksman-Caldentey K., Poutanen K. (2002). Differences between sensory profiles and development of rancidity during long-term storage of native and processed oat. Cereal Chem..

[28] Franklin L.M., Mitchell A.E. (2019). Review of the Sensory and Chemical Characteristics of Almond (*Prunus dulcis*) Flavor. J. Agric. Food Chem..

[29] Moin M., Bommineni P.R., Tyagi W. (2024). Exploration of the pearl millet phospholipase gene family to identify potential candidates for grain quality traits. BMC Genom..

[30] Ghorbani Gorji S., Calingacion M., Smyth H.E., Fitzgerald M. (2019). Effect of natural antioxidants on lipid oxidation in mayonnaise compared with BHA, the industry standard. Metabolomics.

[31] Taylor J.R. (2019). Sorghum and Millets: Taxonomy, History, Distribution, and Production. Sorghum and Millets.

[32] Sharma M., Yadav D.N., Singh A.K., Tomar S.K. (2015). Rheological and functional properties of heat moisture treated pearl millet starch. J. Food Sci. Technol..

[33] Nakayama Y., Saio K., Kito M. (1981). Decomposition of phospholipids in soybean during storage. Cereal Chem..

[34] List G., Mounts T., Lanser A. (1992). Factors promoting the formation of nonhydratable soybean phosphatides. J. Am. Oil Chem..

[35] Kaur A., Neelam K., Kaur K., Kitazumi A., de Los Reyes B.G., Singh K. (2020). Novel allelic variation in the phospholipase d alpha 1 gene (OsPLDα1) of wild oryza species implies to its low expression in rice bran. Sci. Rep..

[36] Zhang S., Zhang W., Martin J.J.J., Qadri R., Fu X., Feng M., Wei L., Zhang A., Yang C., Cao H. (2023). Differential analysis of transcriptomic and metabolomic of free fatty acid rancidity process in oil palm (*Elaeis guineensis*) fruits of different husk types. Front. Plant Sci..

[37] Panche A.N., Diwan A.D., Chandra S.R. (2016). Flavonoids: An Overview. J. Nutr. Sci..

[38] Simunkova M., Barbierikova Z., Jomova K., Hudecova L., Lauro P., Alwasel S.H., Alhazza I., Rhodes C.J., Valko M. (2021). Antioxidant vs. prooxidant properties of the flavonoid, kaempferol, in the Presence of Cu (II) Ions: A ROS-scavenging activity, Fenton reaction and DNA damage study. Int. J. Mol. Sci..

[39] Chaaban H., Ioannou I., Paris C., Charbonnel C., Ghoul M. (2017). The photostability of flavanones, flavonols and flavones and evolution of their antioxidant activity. J. Photochem. Photobiol. A.

