# Supplementary material for: Comparative Metabolomics to Unravel the Biochemical Mechanism Associated with Rancidity in Pearl Millet (Pennisetum glaucum L.)
Source: Int J Mol Sci. 2024 Oct 29;25(21):11583. doi: 10.3390/ijms252111583 (PMC11547105; doi:10.3390/ijms252111583)

## Slide 1
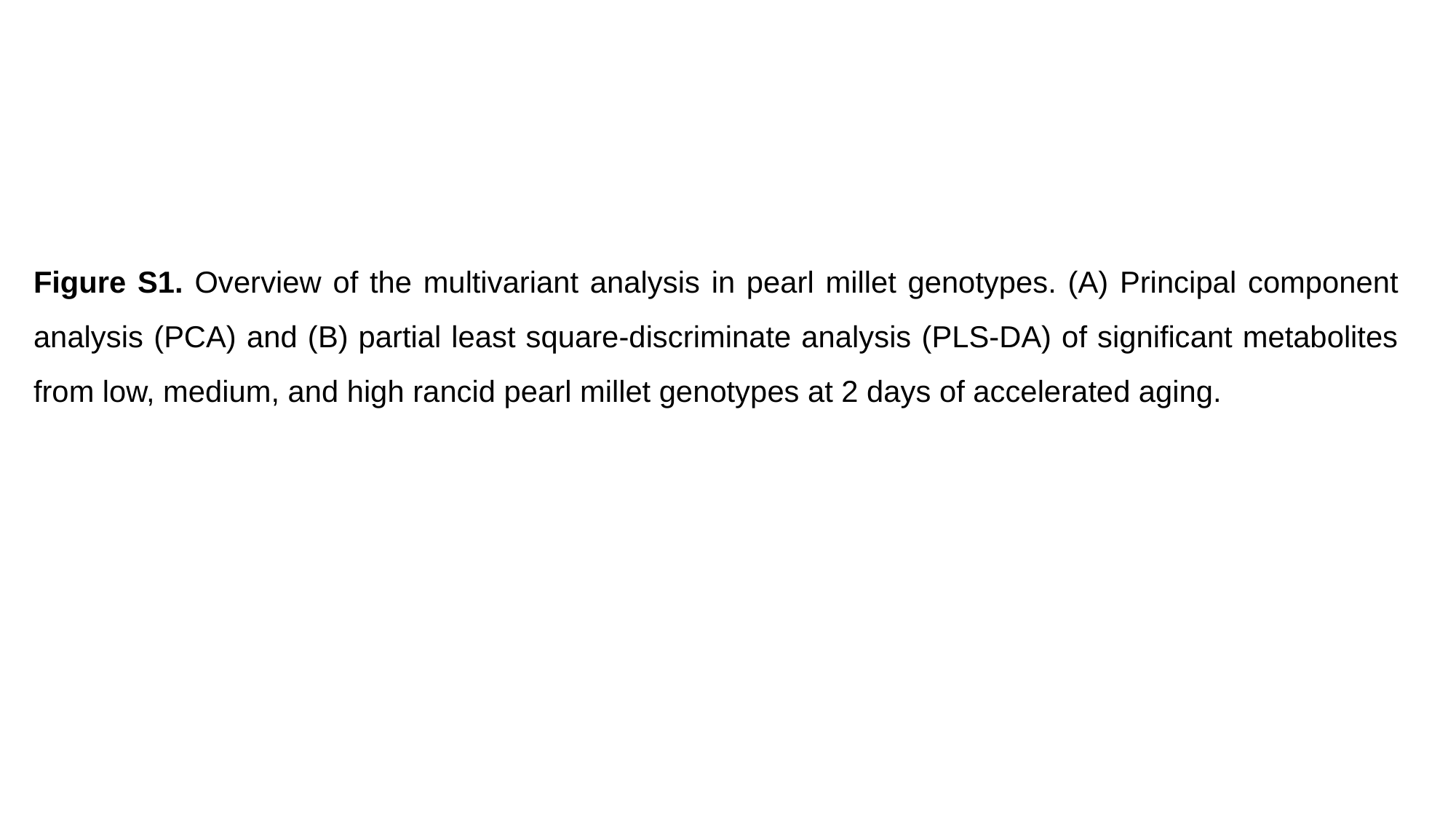

Figure S1. Overview of the multivariant analysis in pearl millet genotypes. (A) Principal component analysis (PCA) and (B) partial least square-discriminate analysis (PLS-DA) of significant metabolites from low, medium, and high rancid pearl millet genotypes at 2 days of accelerated aging.

## Slide 2
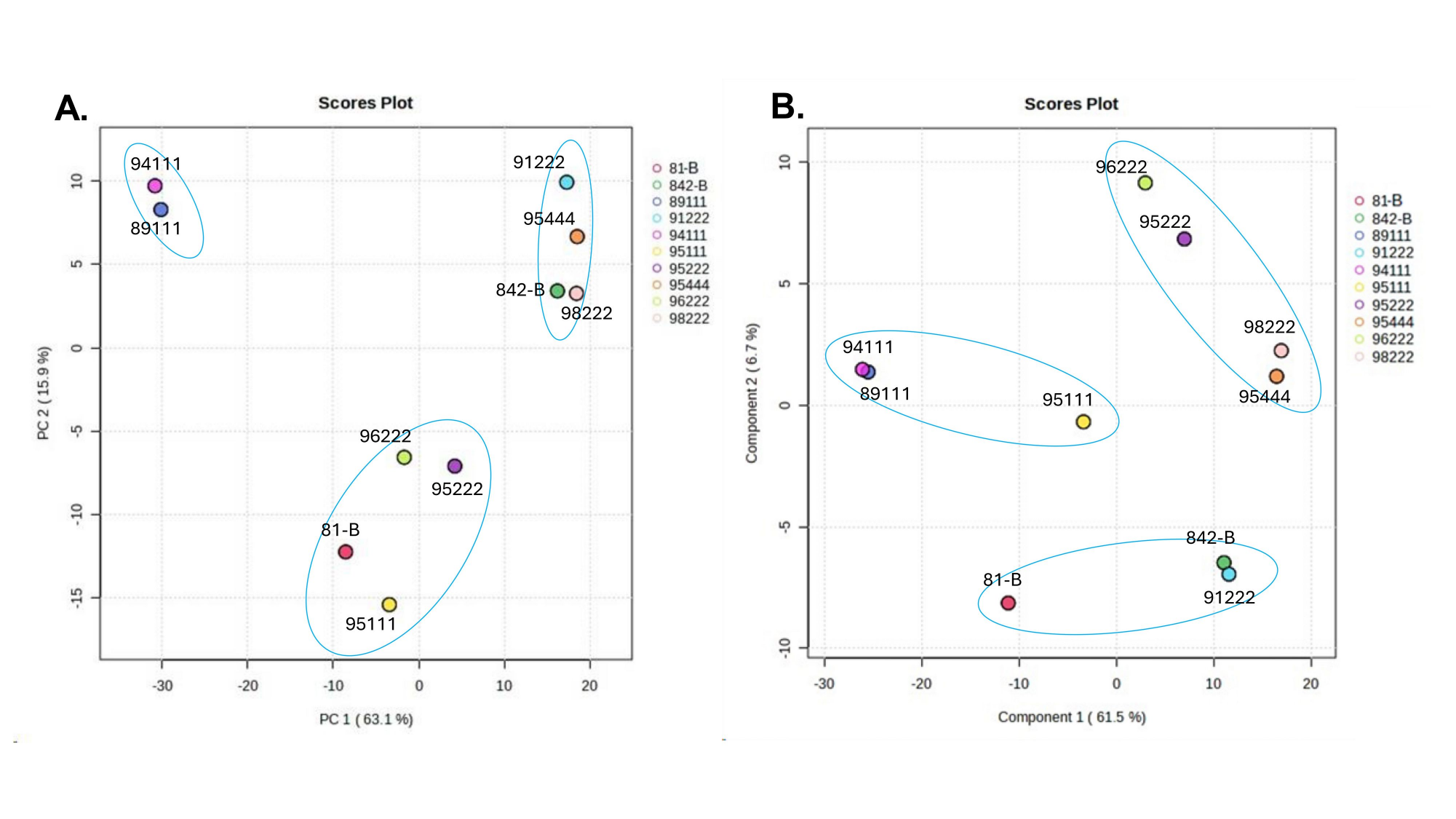

## Slide 3
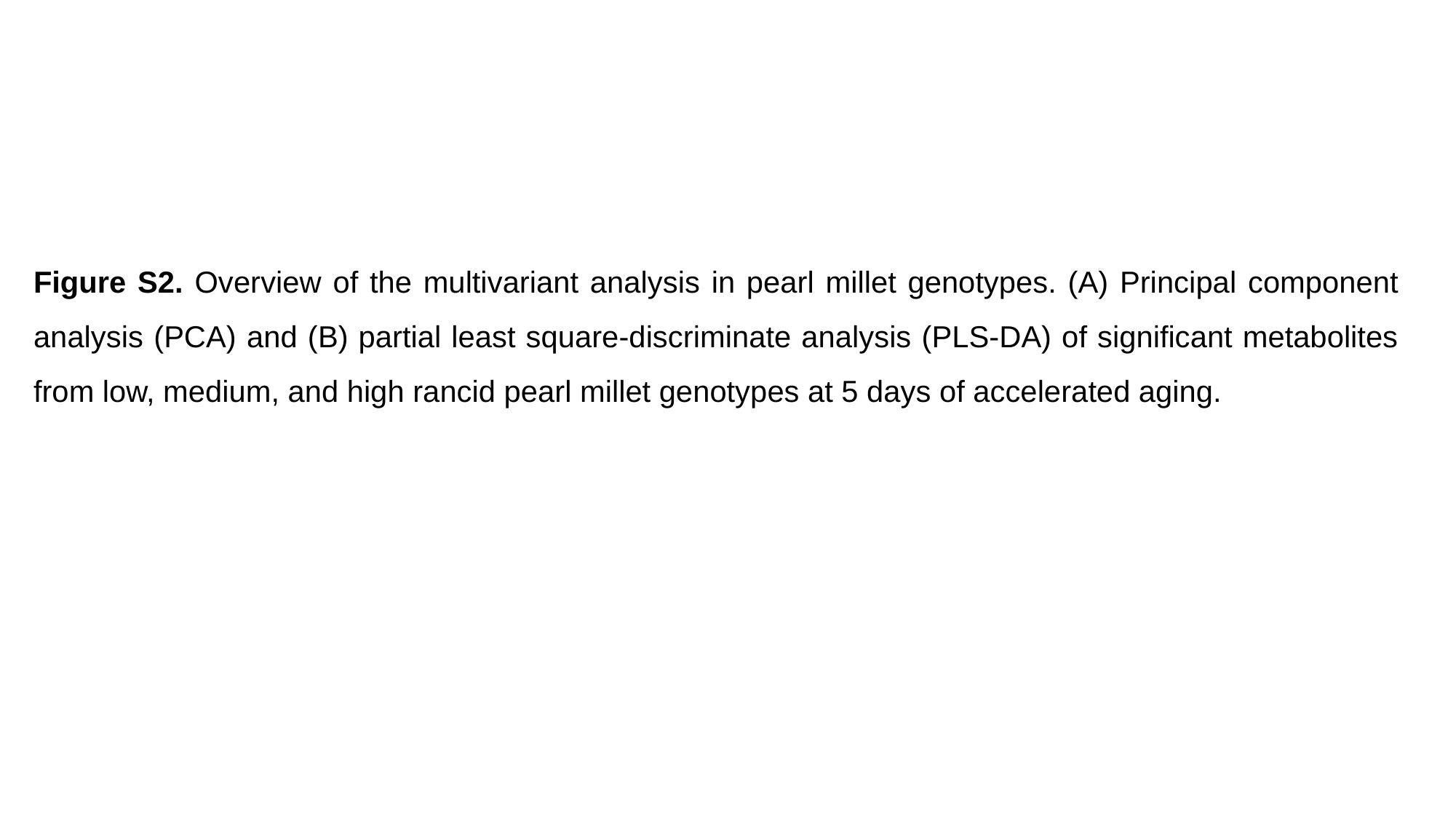

Figure S2. Overview of the multivariant analysis in pearl millet genotypes. (A) Principal component analysis (PCA) and (B) partial least square-discriminate analysis (PLS-DA) of significant metabolites from low, medium, and high rancid pearl millet genotypes at 5 days of accelerated aging.

## Slide 4
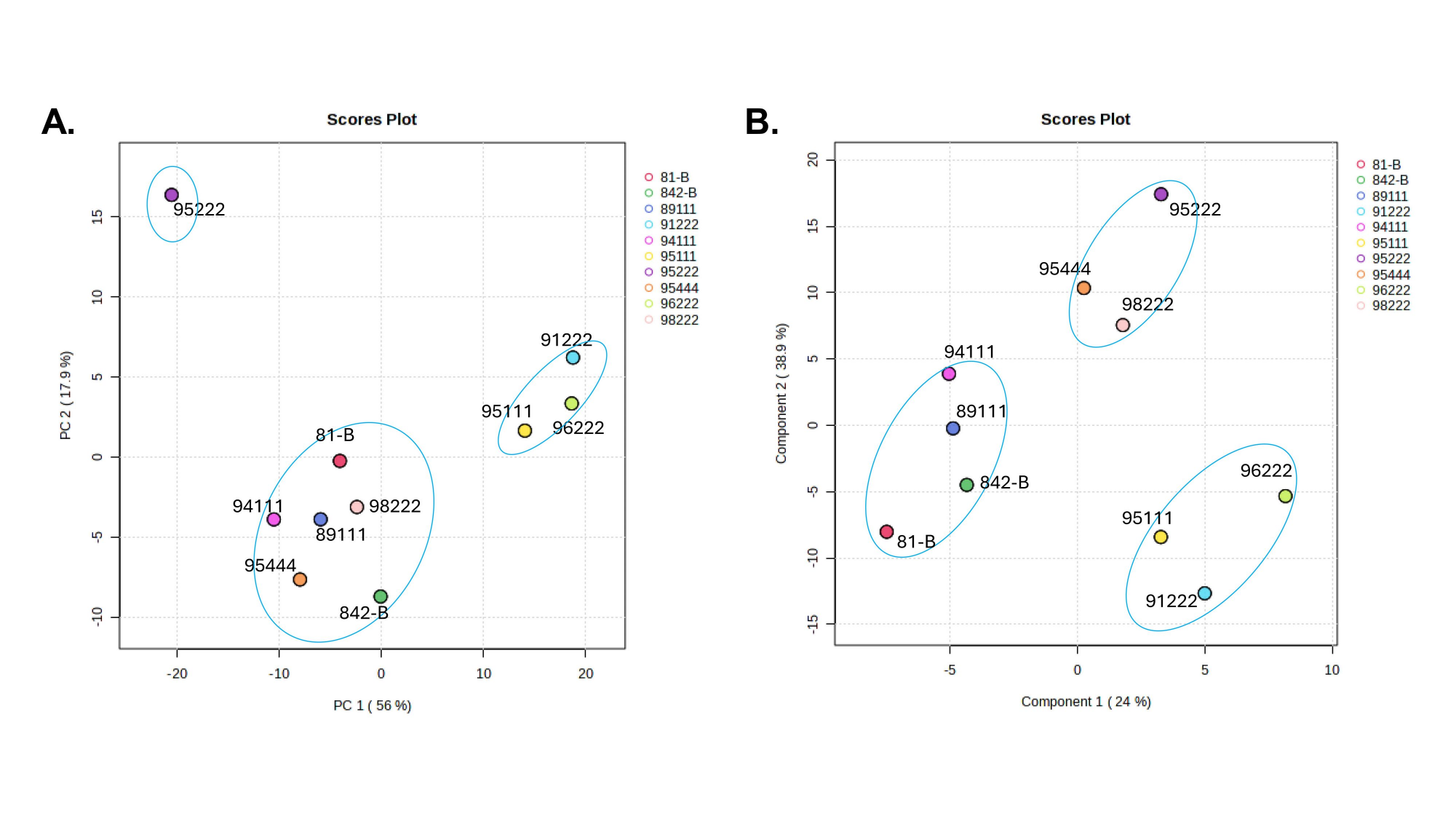

## Slide 5
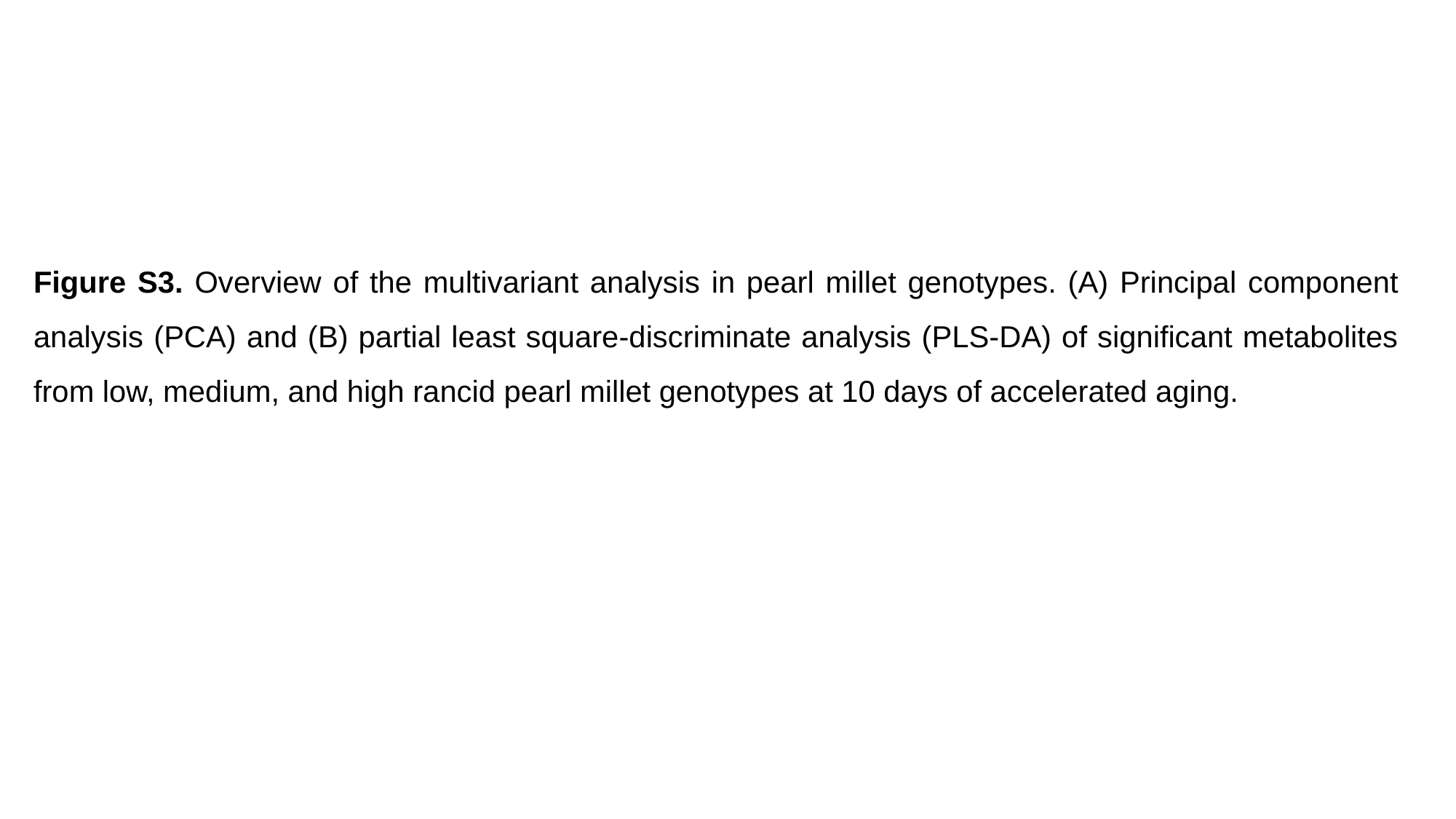

Figure S3. Overview of the multivariant analysis in pearl millet genotypes. (A) Principal component analysis (PCA) and (B) partial least square-discriminate analysis (PLS-DA) of significant metabolites from low, medium, and high rancid pearl millet genotypes at 10 days of accelerated aging.

## Slide 6
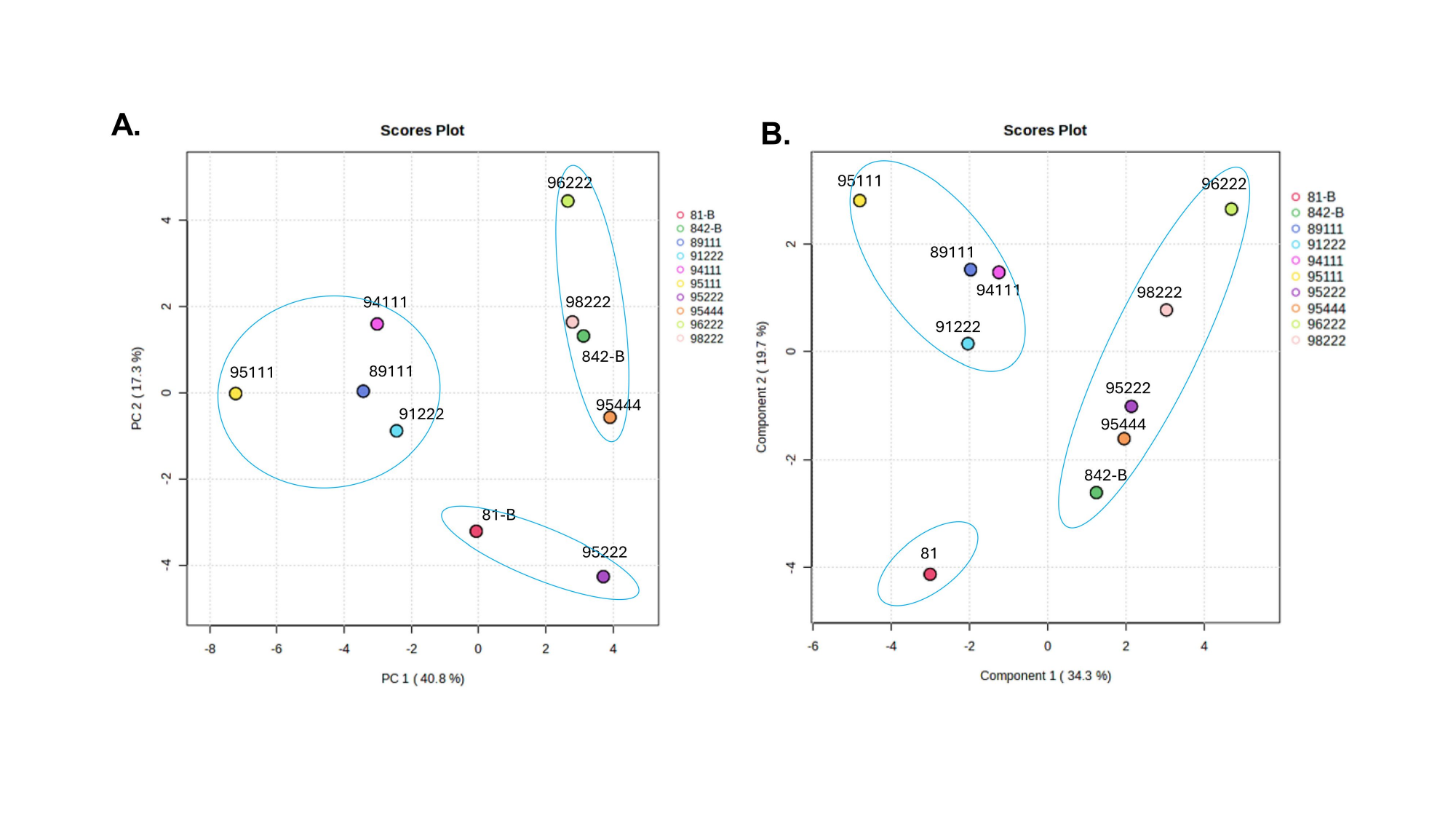

## Slide 7
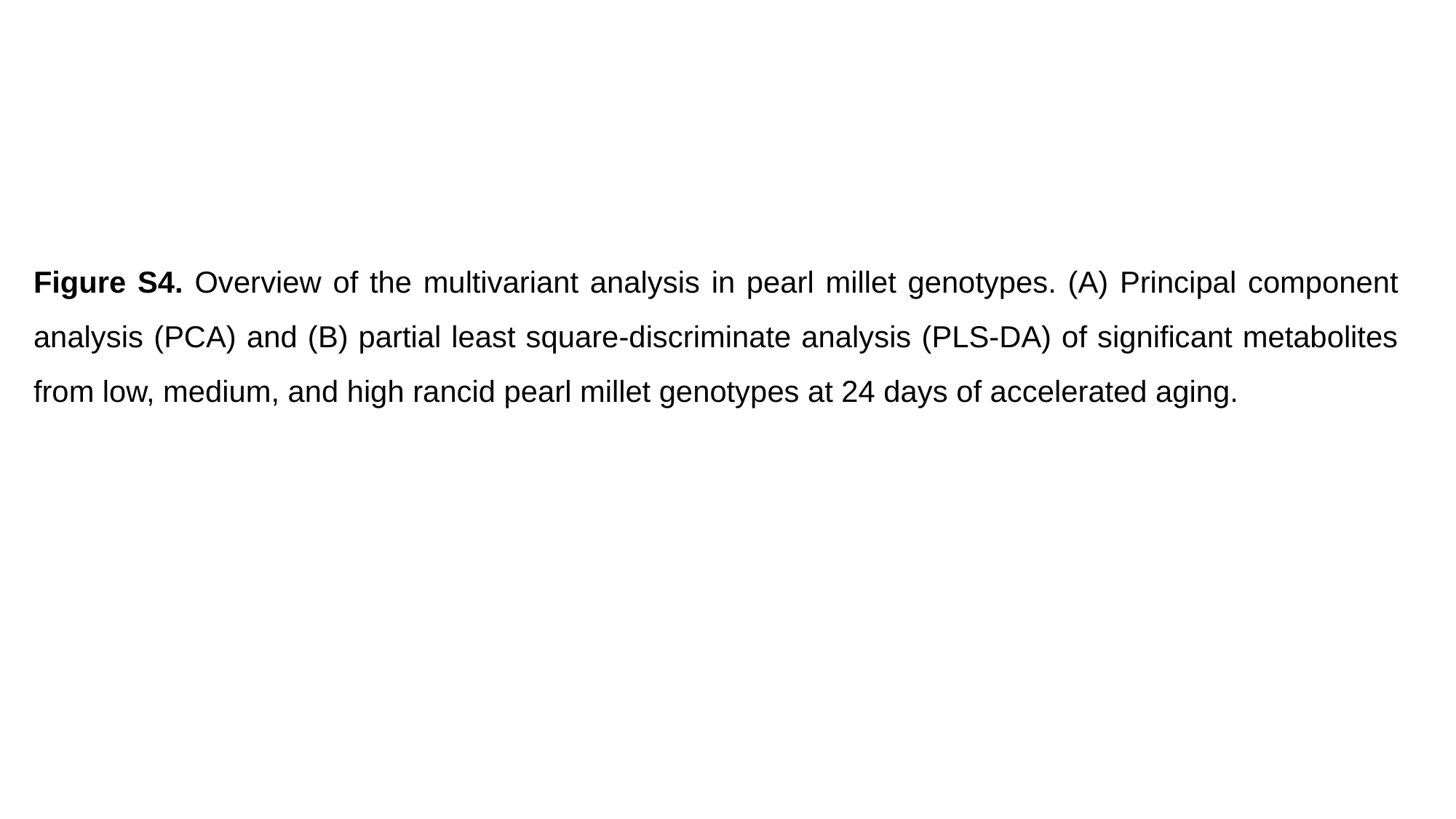

Figure S4. Overview of the multivariant analysis in pearl millet genotypes. (A) Principal component analysis (PCA) and (B) partial least square-discriminate analysis (PLS-DA) of significant metabolites from low, medium, and high rancid pearl millet genotypes at 24 days of accelerated aging.

## Slide 8
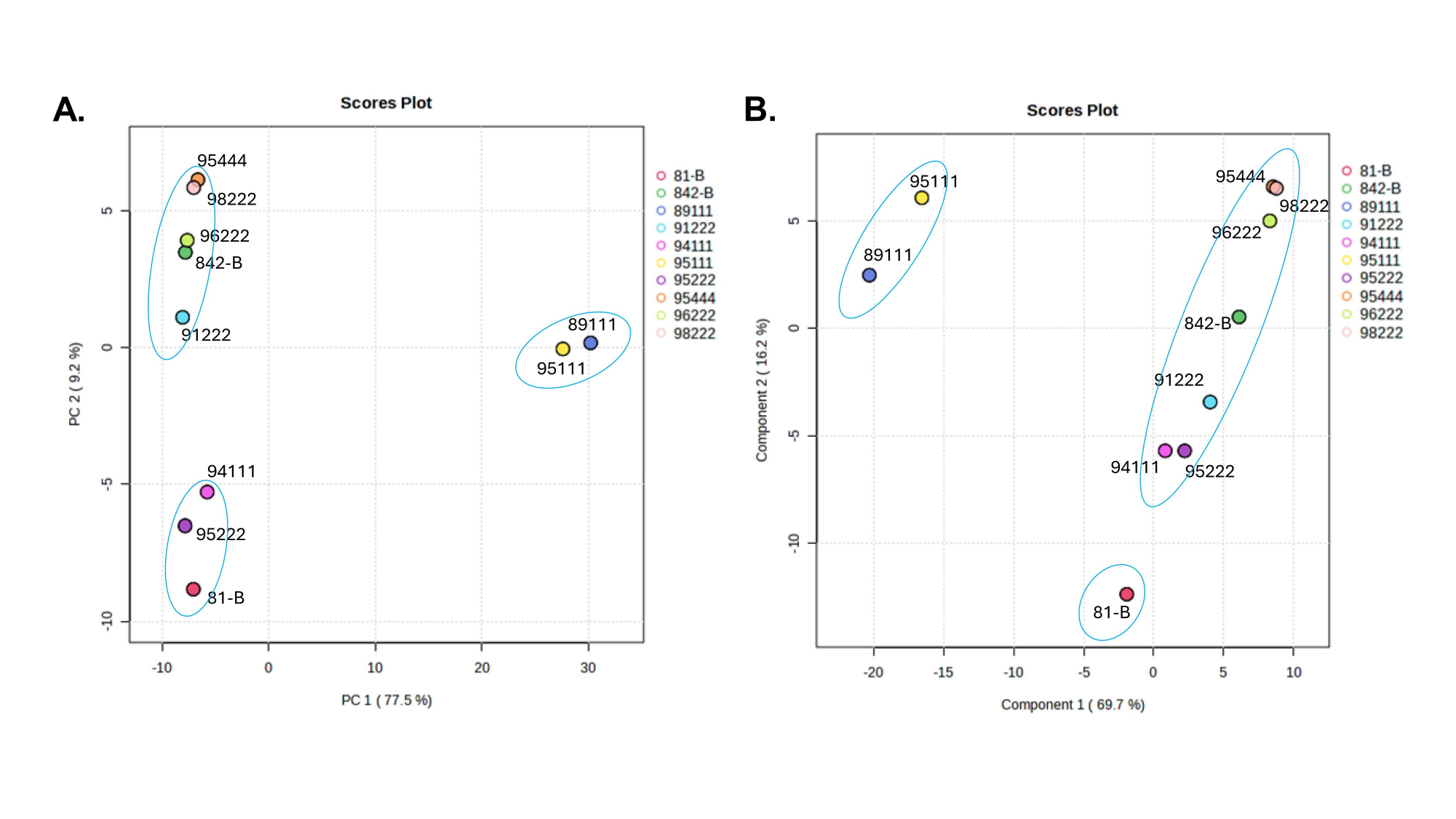

## Slide 9
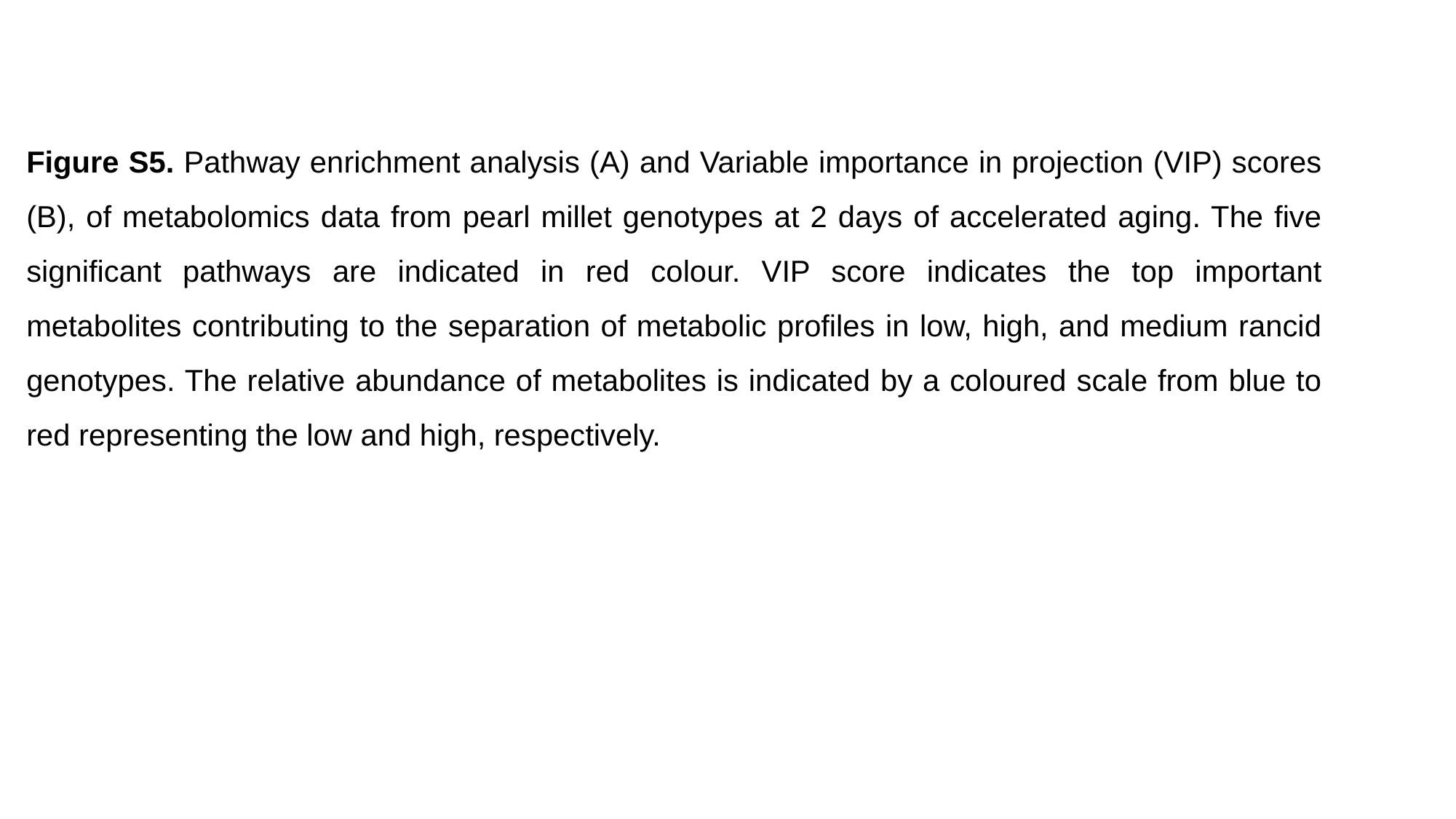

Figure S5. Pathway enrichment analysis (A) and Variable importance in projection (VIP) scores (B), of metabolomics data from pearl millet genotypes at 2 days of accelerated aging. The five significant pathways are indicated in red colour. VIP score indicates the top important metabolites contributing to the separation of metabolic profiles in low, high, and medium rancid genotypes. The relative abundance of metabolites is indicated by a coloured scale from blue to red representing the low and high, respectively.

## Slide 10
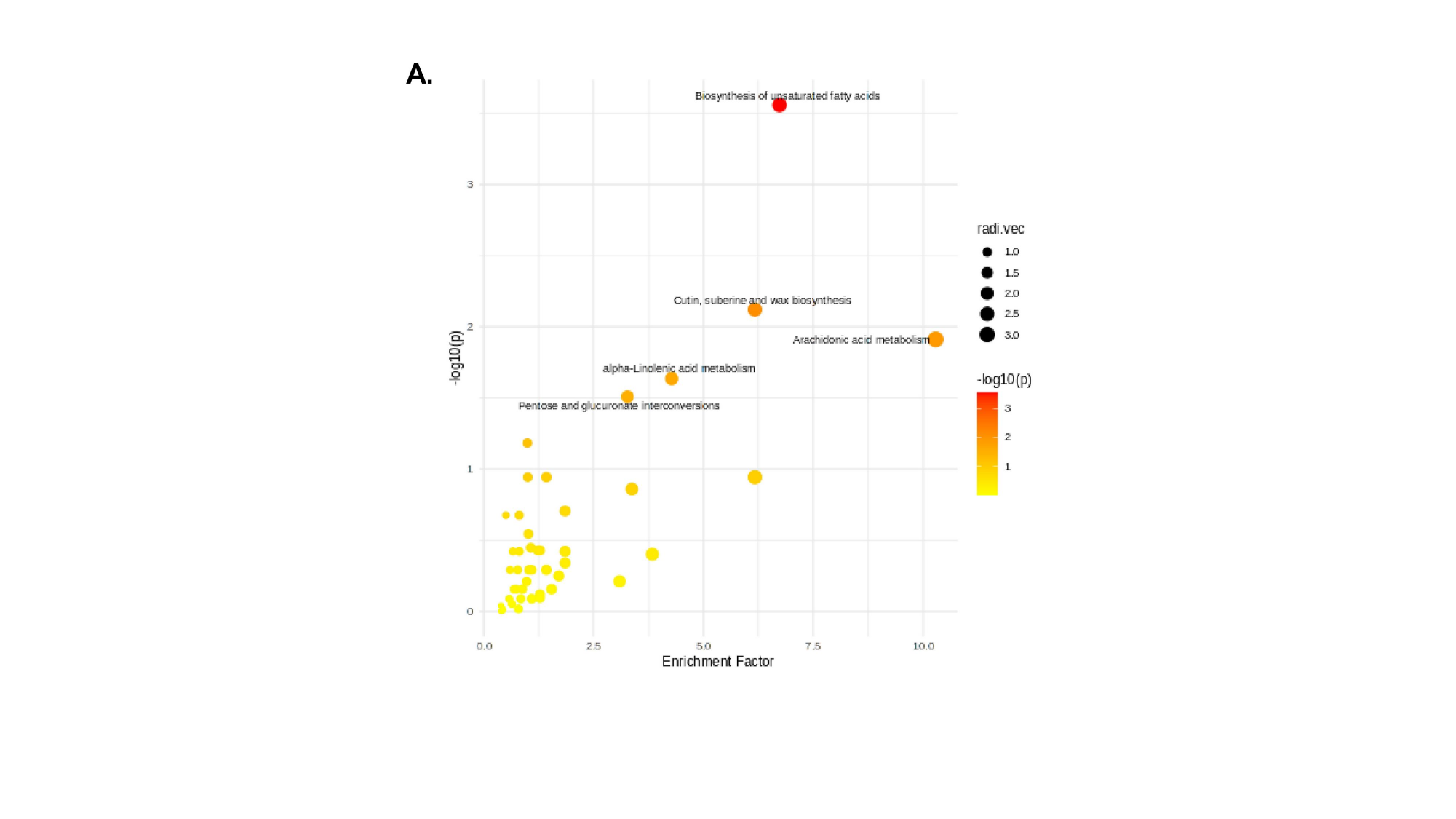

## Slide 11
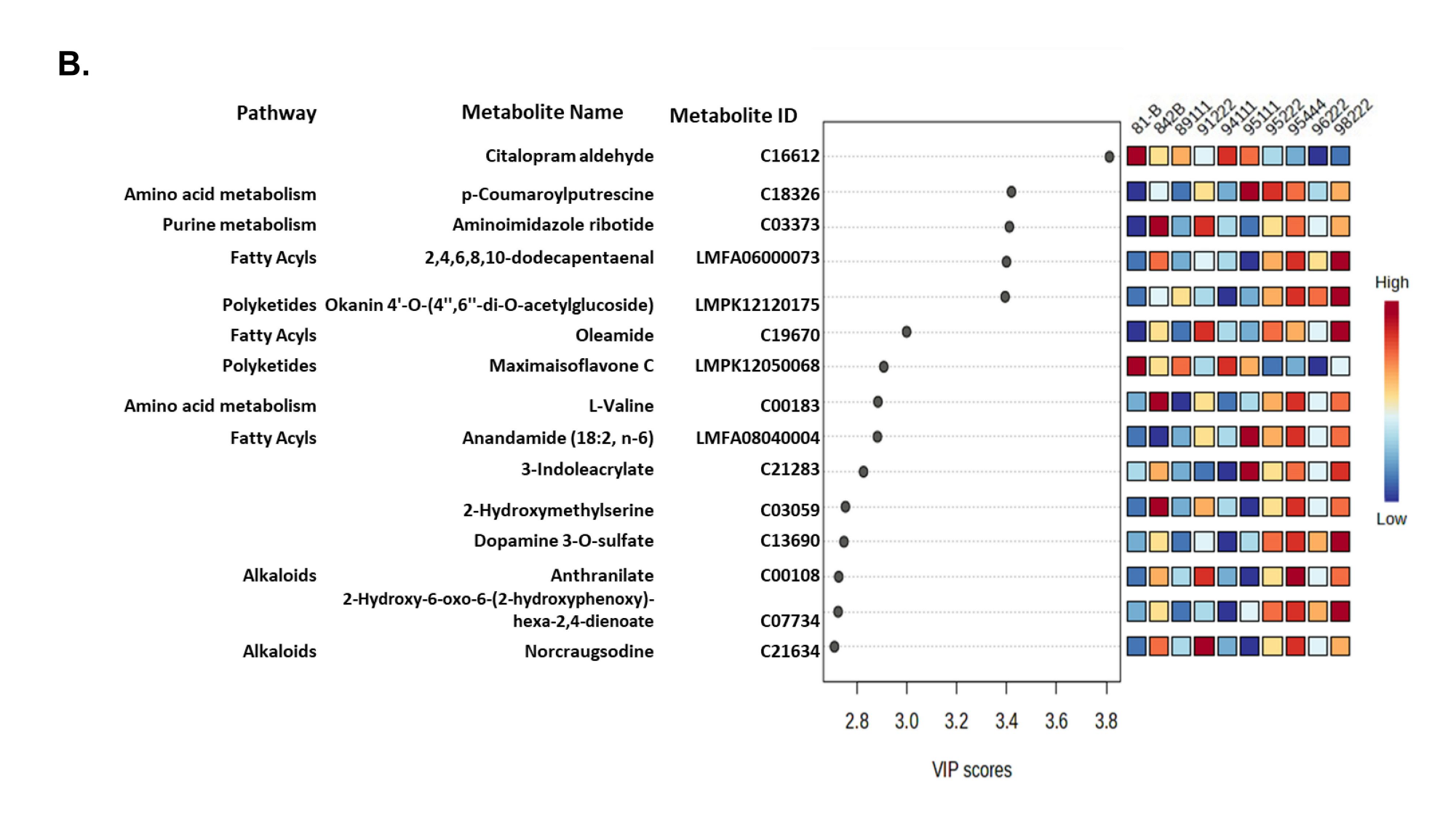

## Slide 12
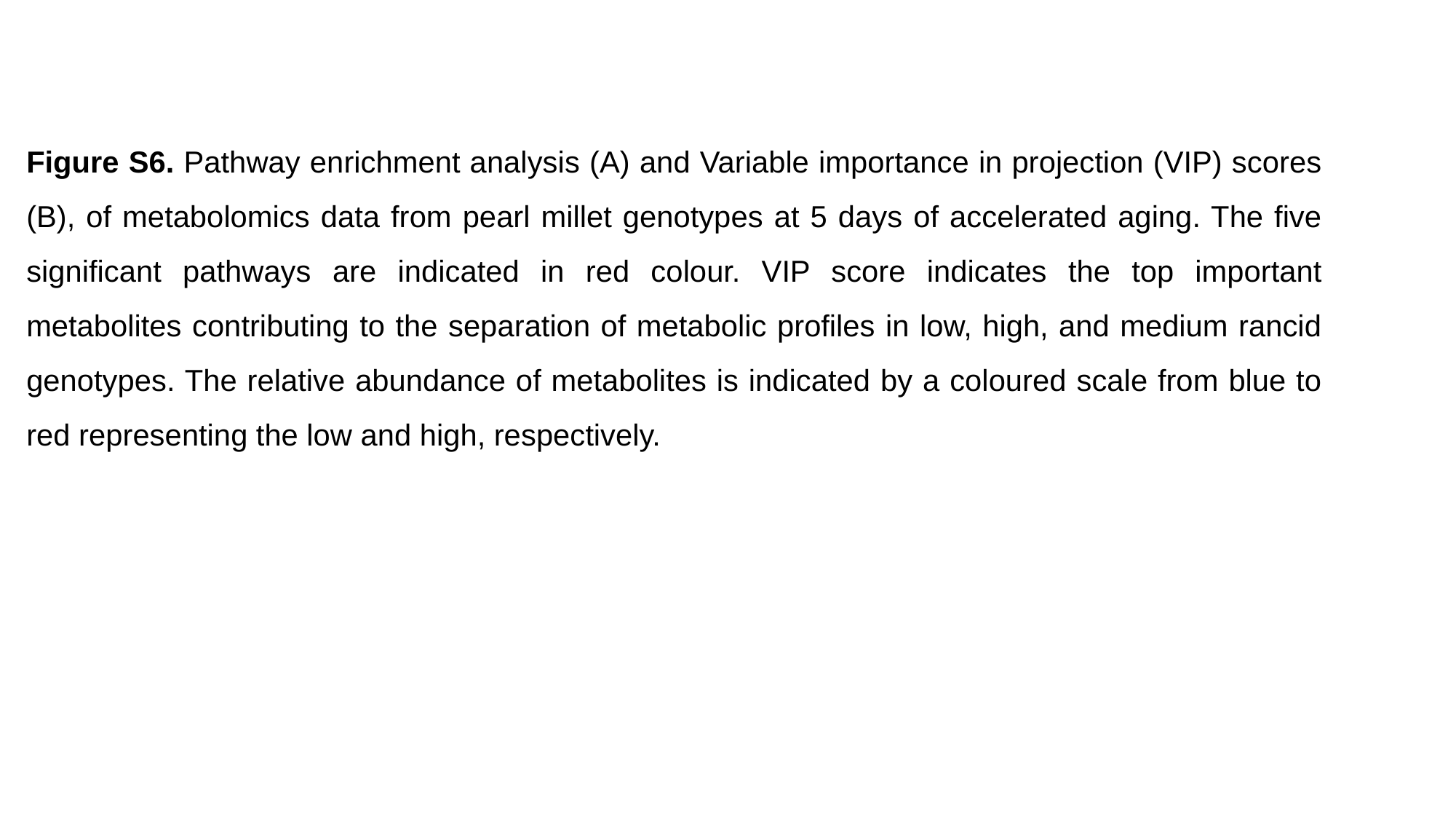

Figure S6. Pathway enrichment analysis (A) and Variable importance in projection (VIP) scores (B), of metabolomics data from pearl millet genotypes at 5 days of accelerated aging. The five significant pathways are indicated in red colour. VIP score indicates the top important metabolites contributing to the separation of metabolic profiles in low, high, and medium rancid genotypes. The relative abundance of metabolites is indicated by a coloured scale from blue to red representing the low and high, respectively.

## Slide 13
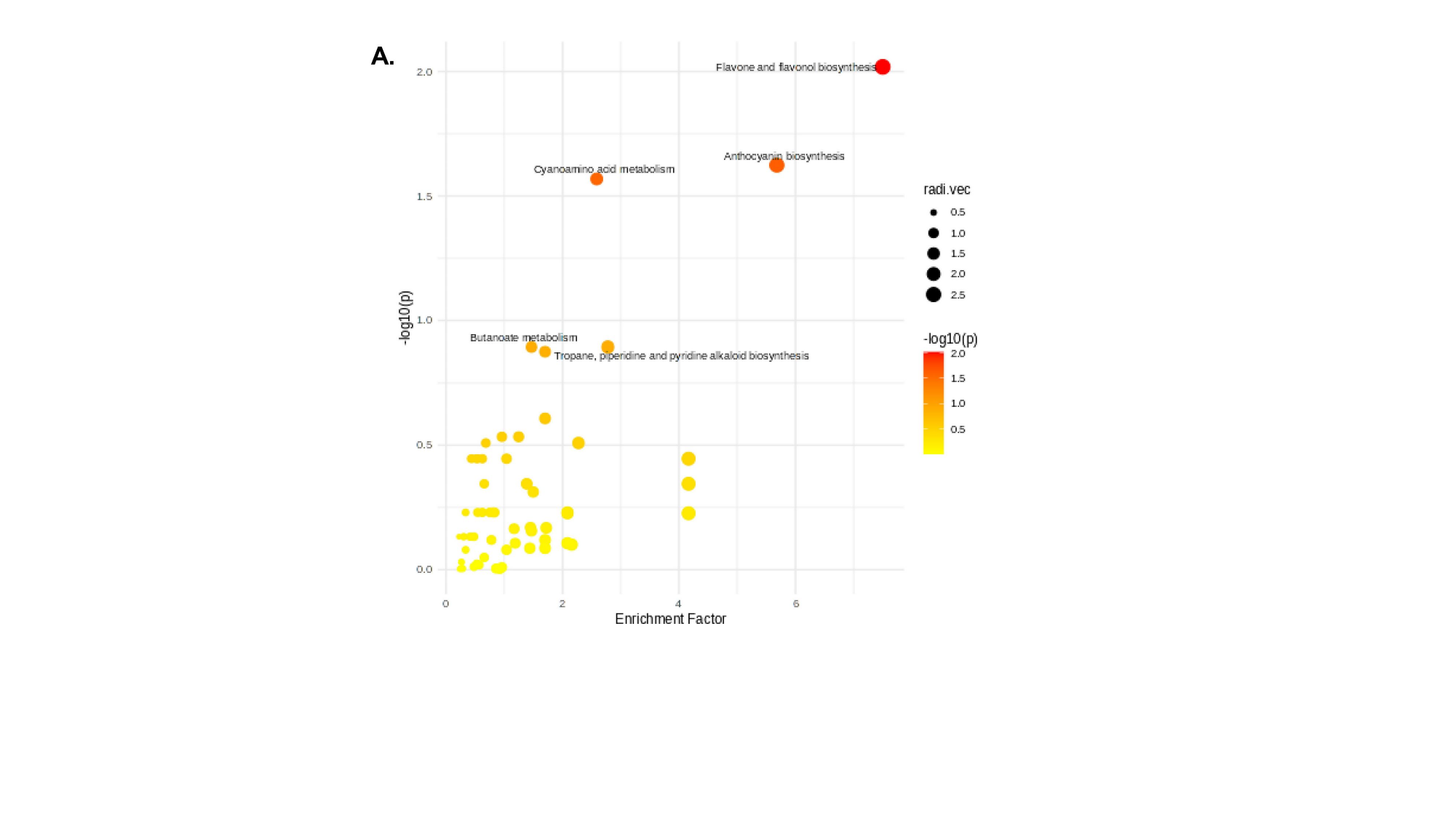

## Slide 14
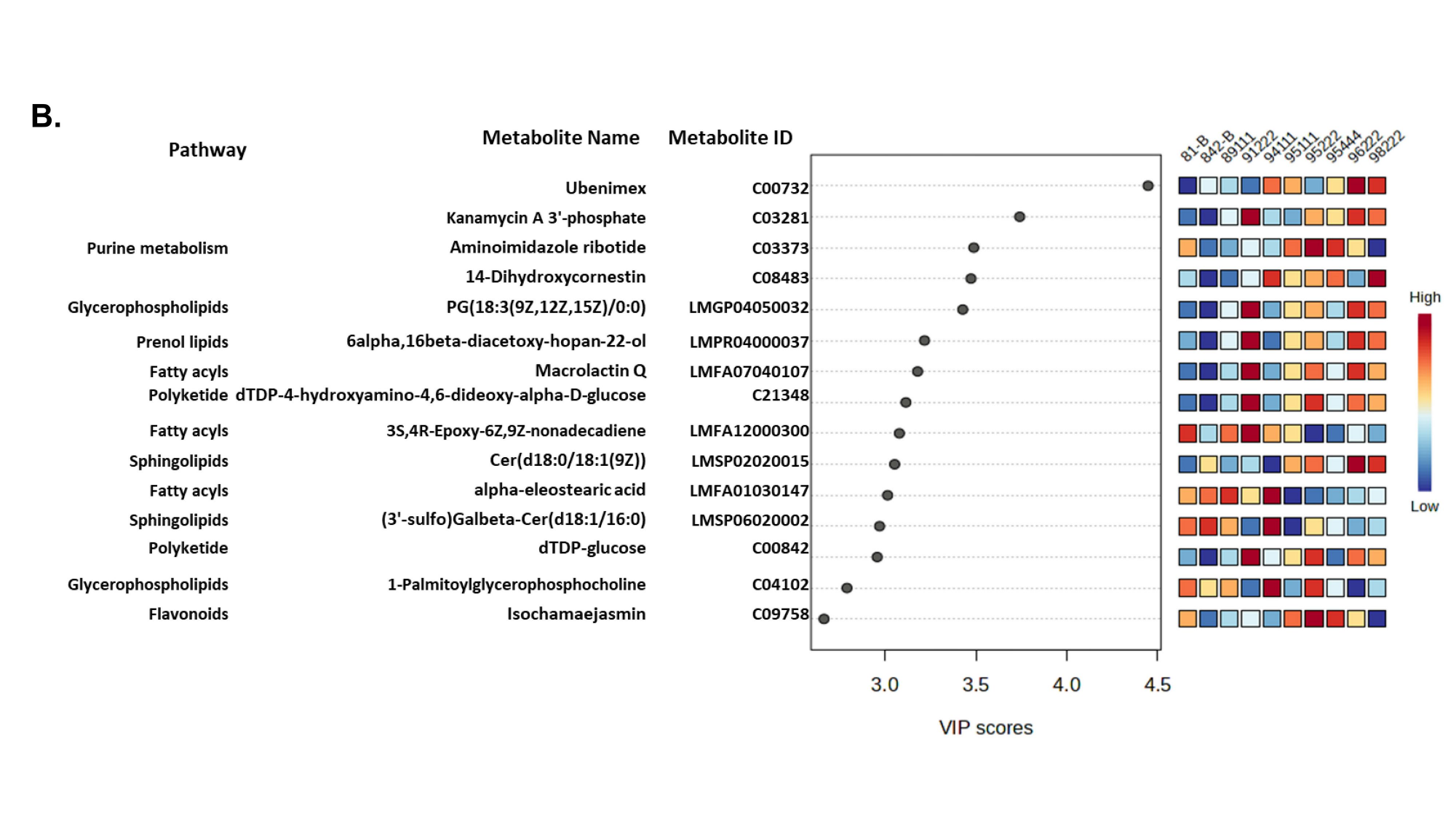

## Slide 15
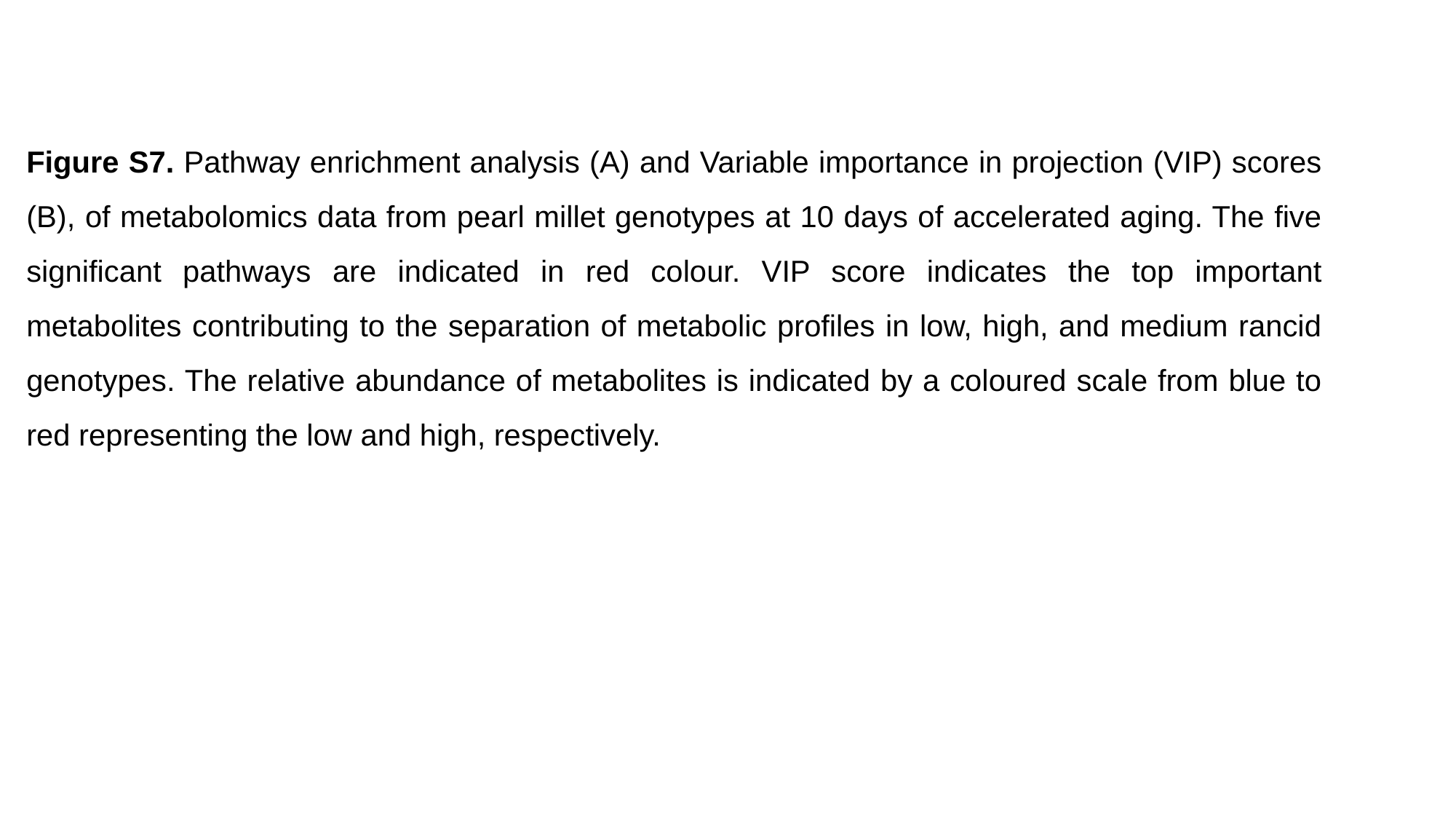

Figure S7. Pathway enrichment analysis (A) and Variable importance in projection (VIP) scores (B), of metabolomics data from pearl millet genotypes at 10 days of accelerated aging. The five significant pathways are indicated in red colour. VIP score indicates the top important metabolites contributing to the separation of metabolic profiles in low, high, and medium rancid genotypes. The relative abundance of metabolites is indicated by a coloured scale from blue to red representing the low and high, respectively.

## Slide 16
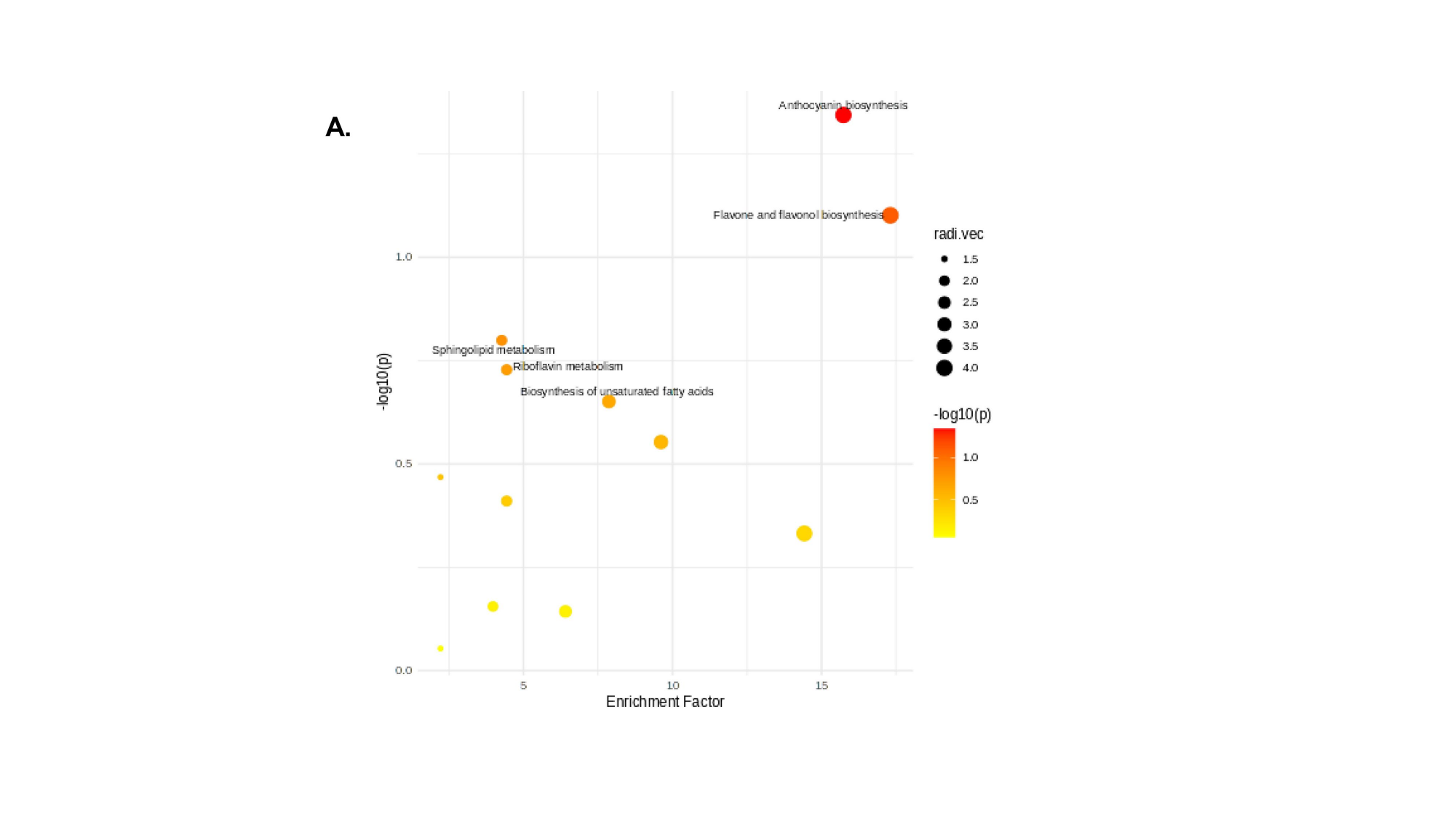

## Slide 17
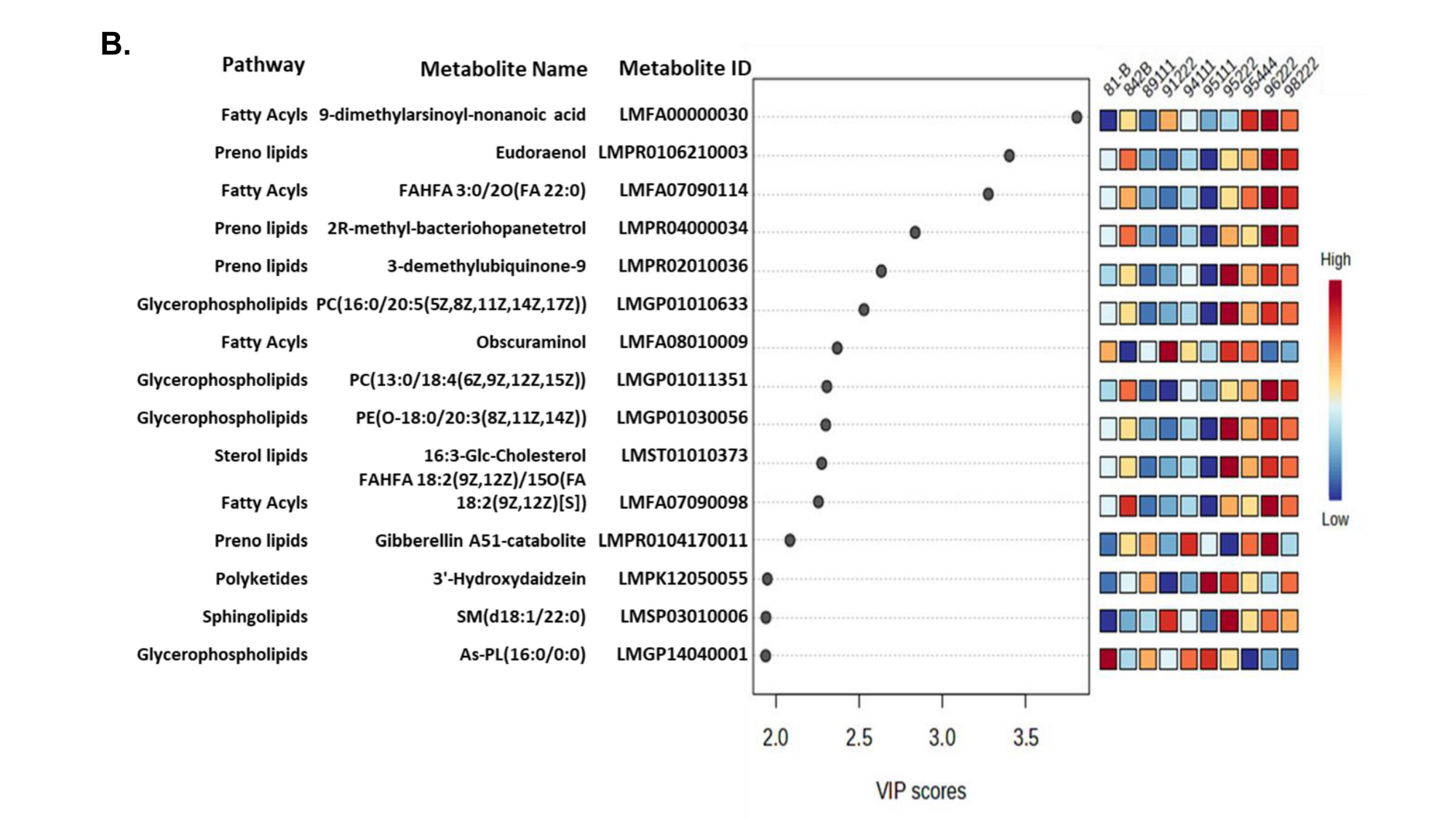

## Slide 18
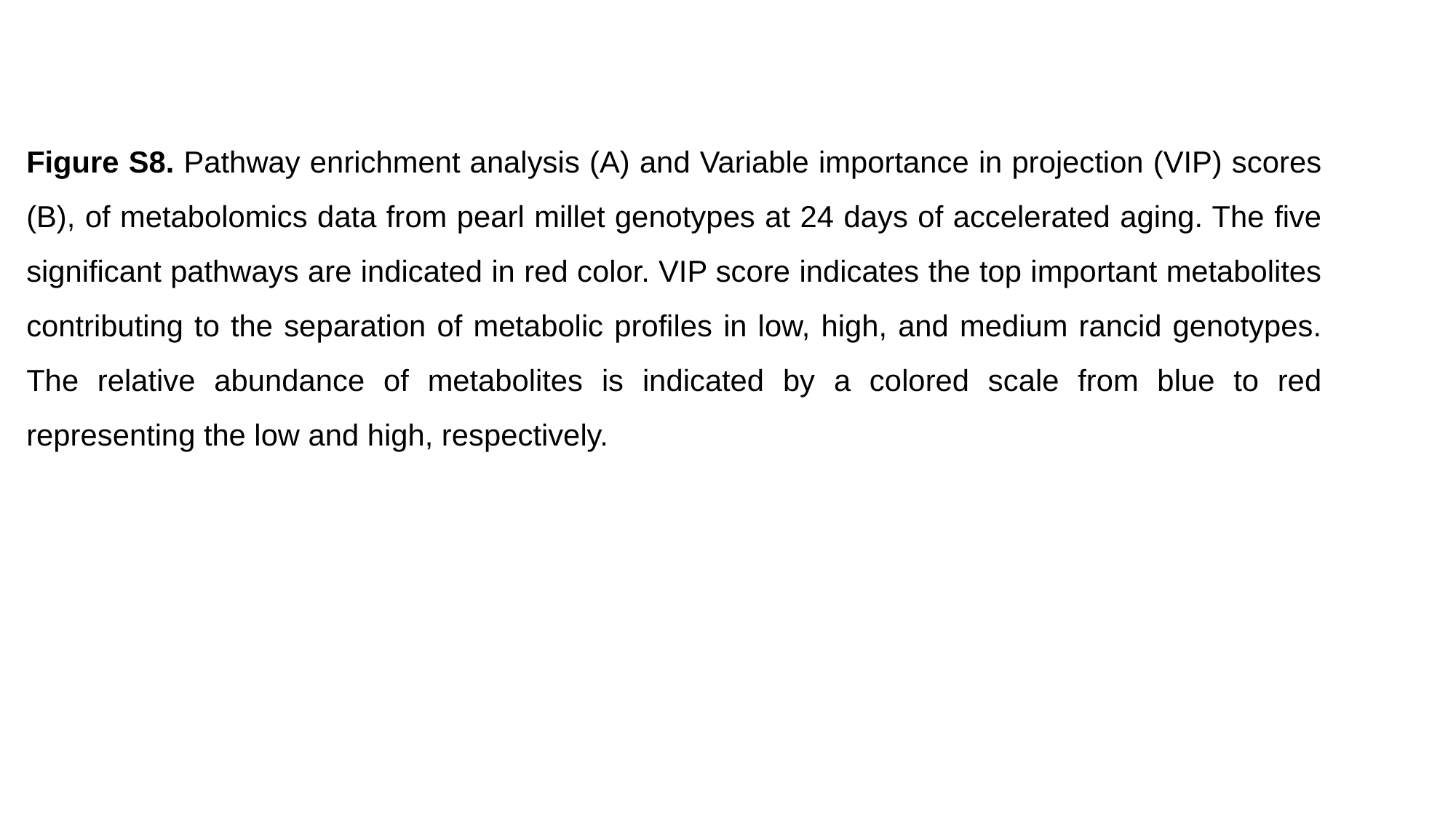

Figure S8. Pathway enrichment analysis (A) and Variable importance in projection (VIP) scores (B), of metabolomics data from pearl millet genotypes at 24 days of accelerated aging. The five significant pathways are indicated in red color. VIP score indicates the top important metabolites contributing to the separation of metabolic profiles in low, high, and medium rancid genotypes. The relative abundance of metabolites is indicated by a colored scale from blue to red representing the low and high, respectively.

## Slide 19
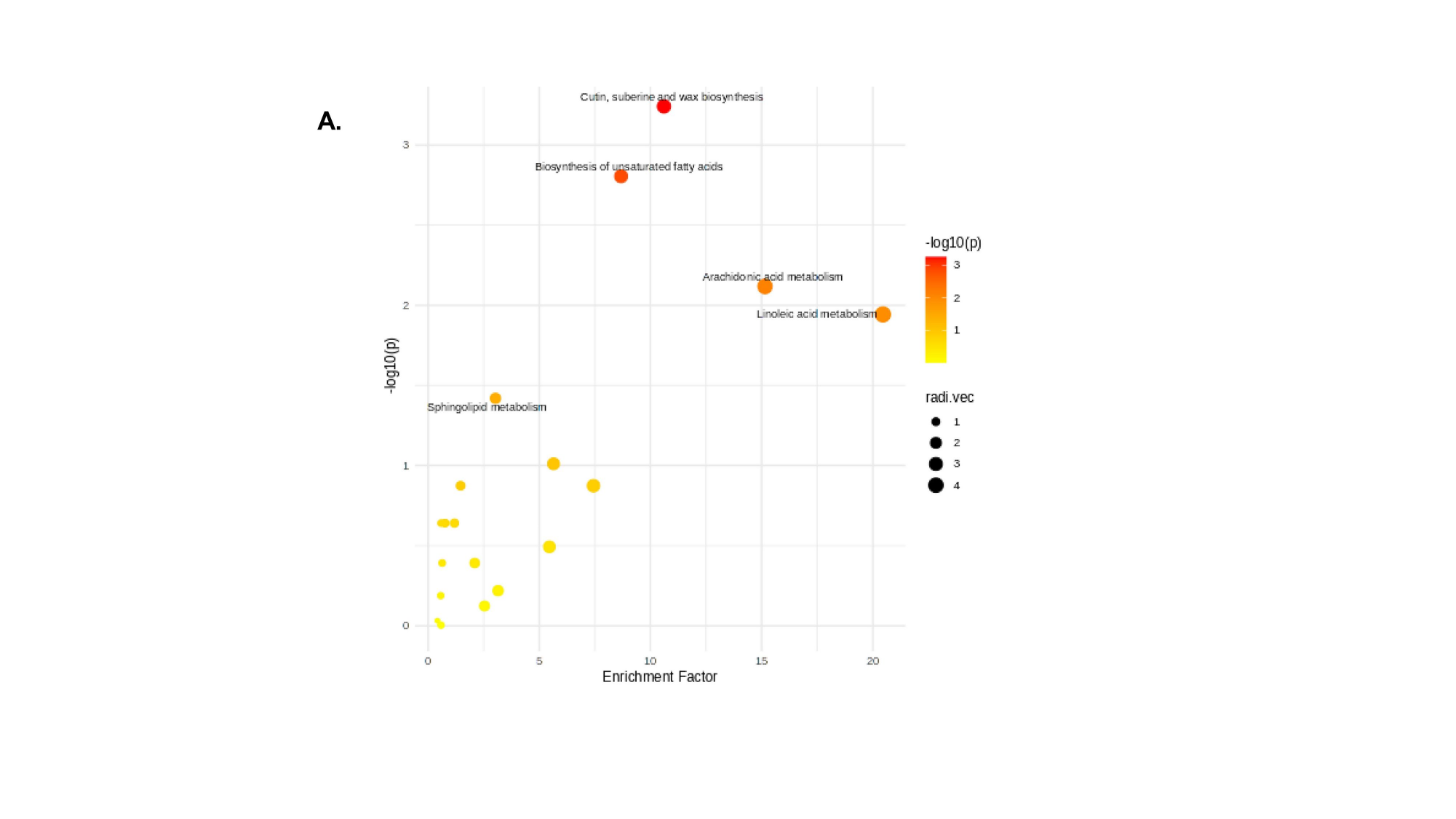

## Slide 20
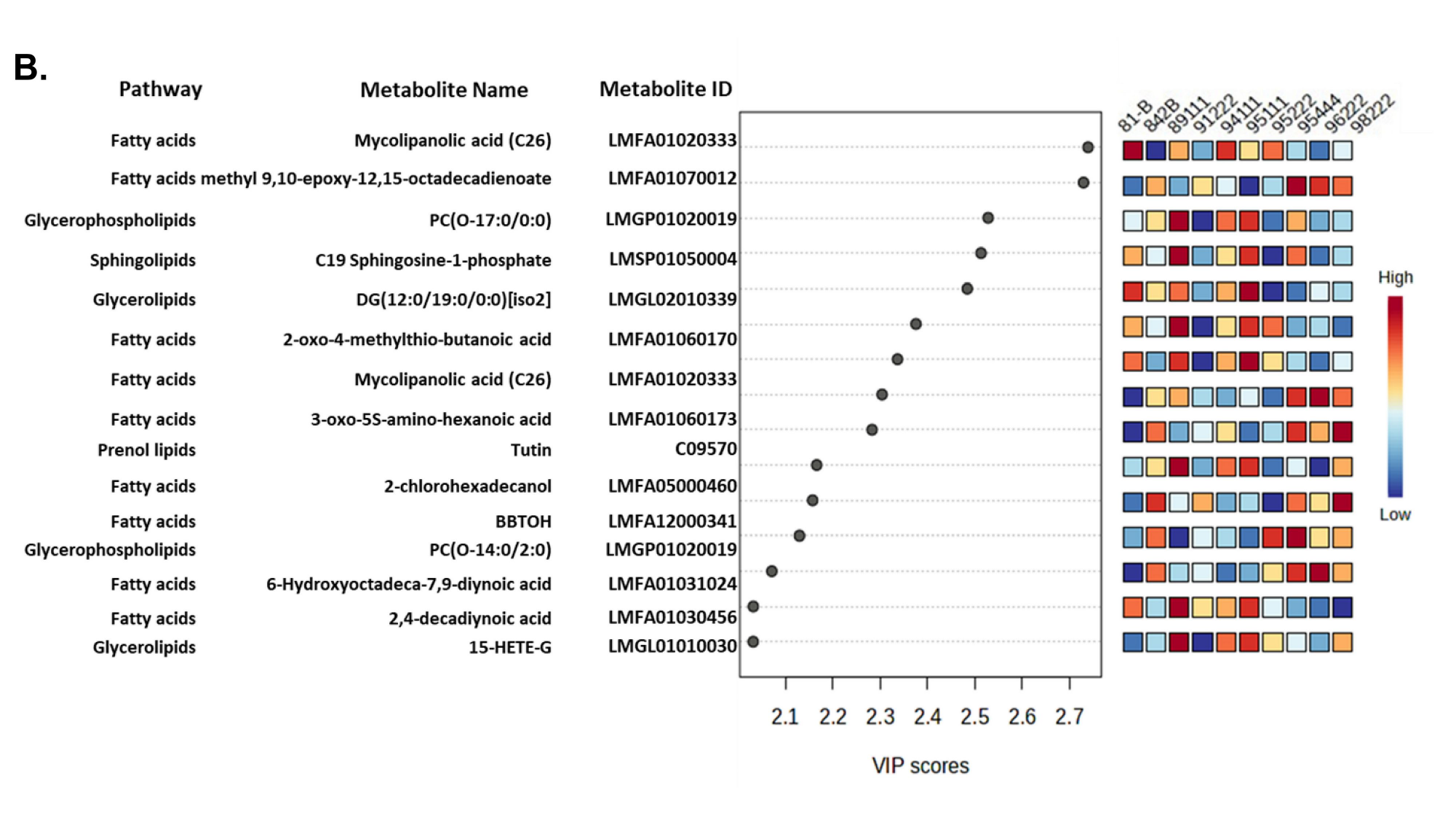

## Slide 21
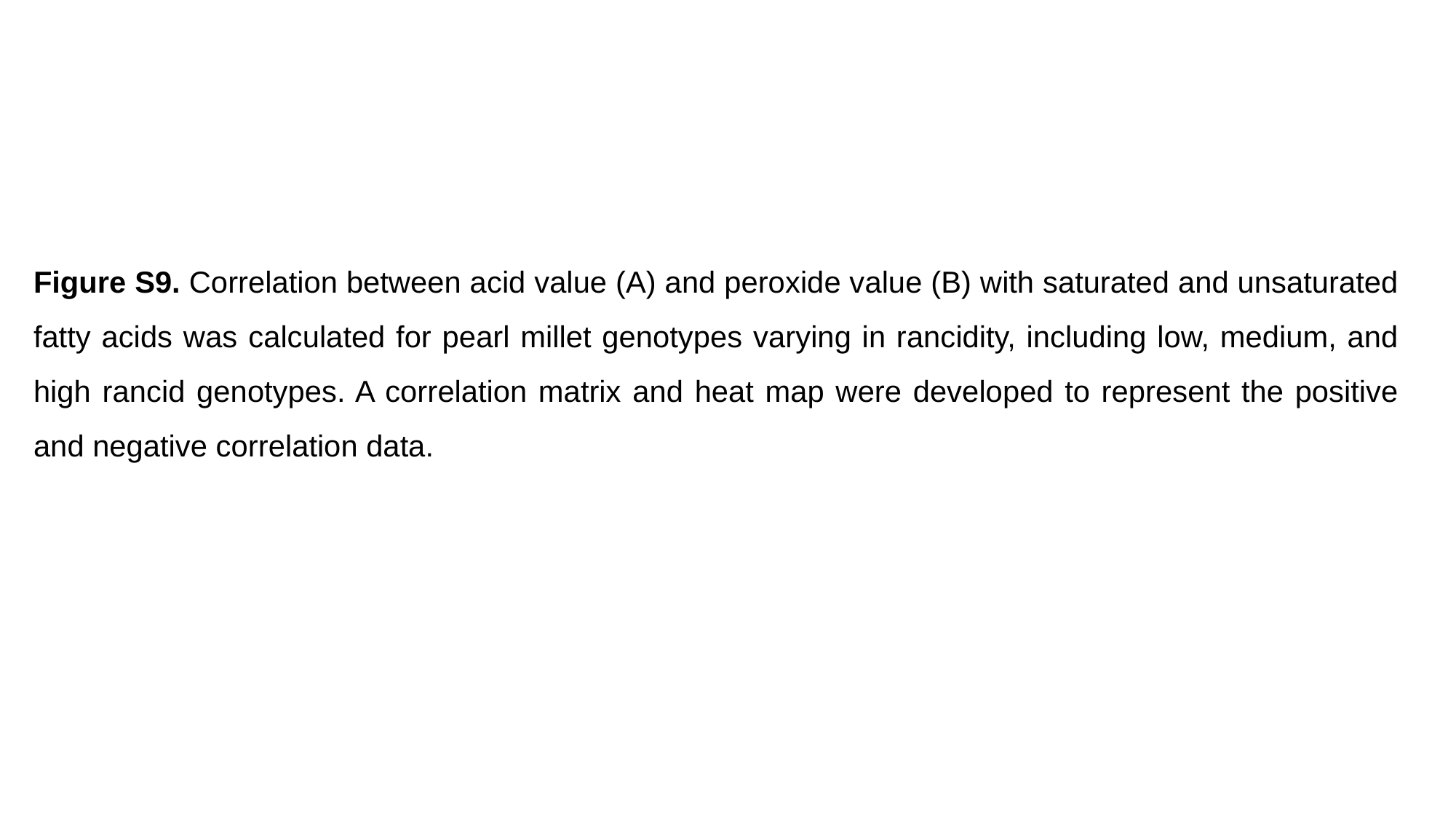

Figure S9. Correlation between acid value (A) and peroxide value (B) with saturated and unsaturated fatty acids was calculated for pearl millet genotypes varying in rancidity, including low, medium, and high rancid genotypes. A correlation matrix and heat map were developed to represent the positive and negative correlation data.

## Slide 22
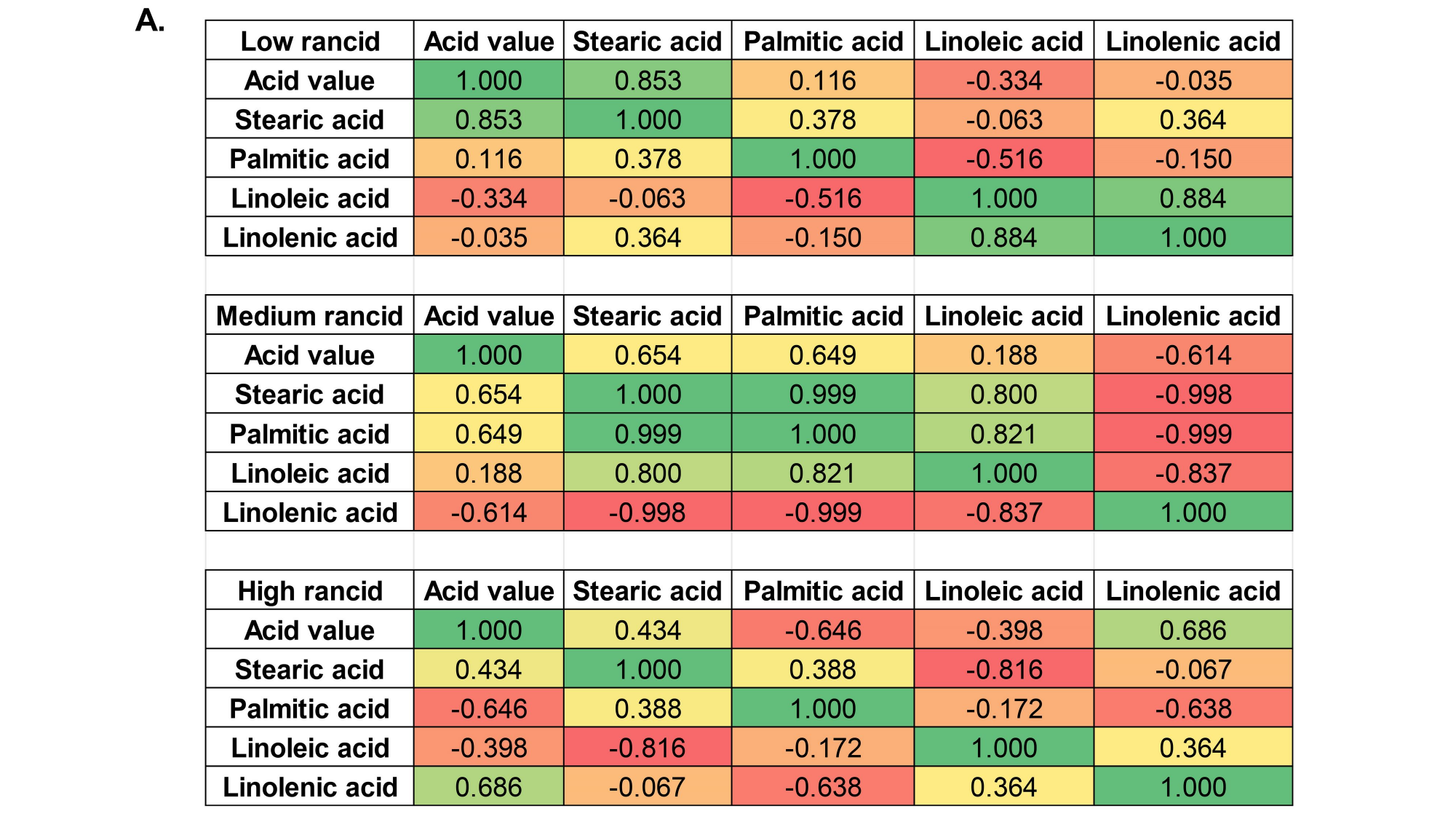

## Slide 23
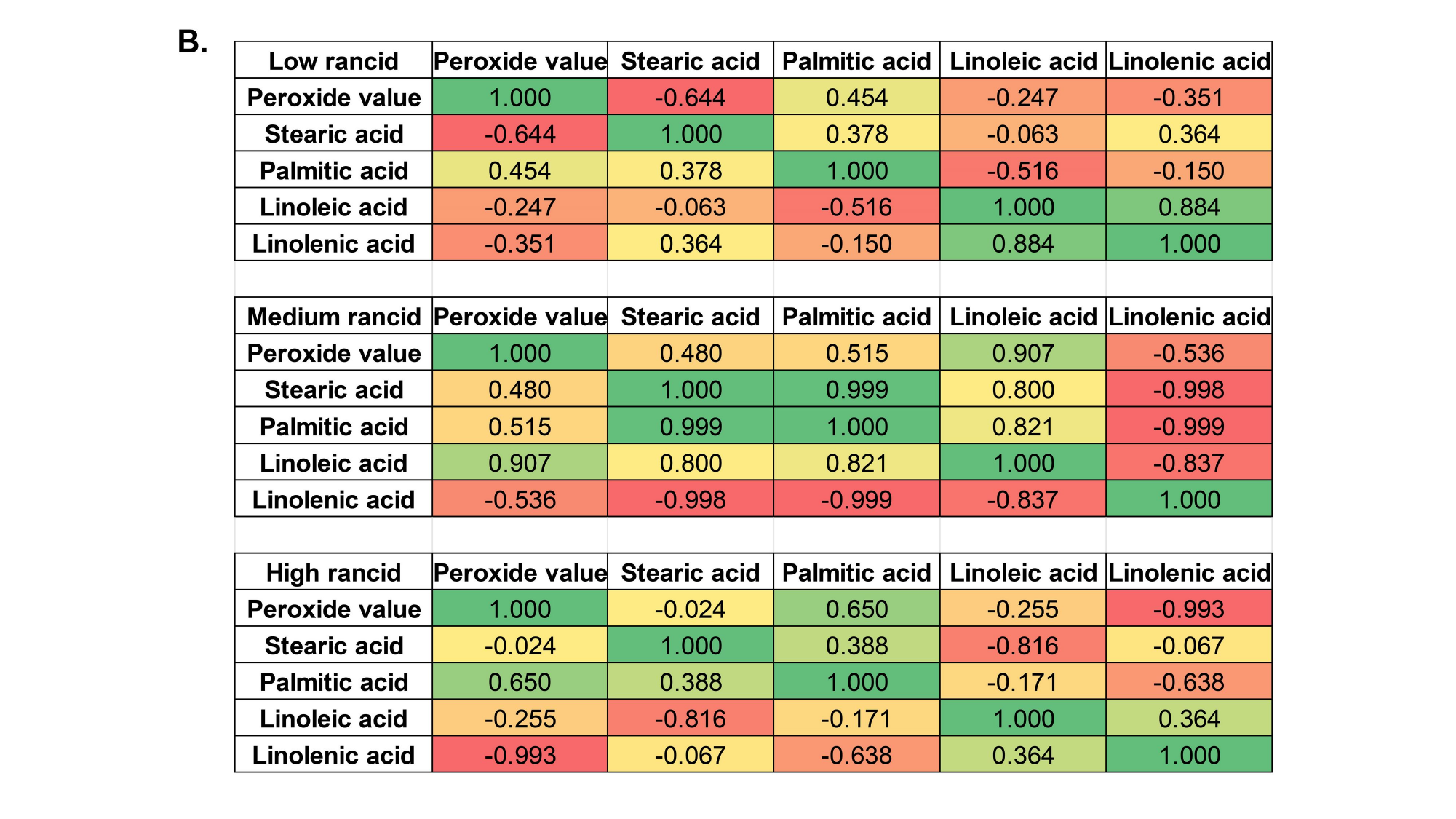

Supplement: Supplementary file 1 [file ijms-25-11583-s001.zip › IJMS_supplementary/Updated_SFigures_Meta.pptx]
